# Current Techniques to Study Beneficial Plant-Microbe Interactions

**DOI:** 10.3390/microorganisms10071380

**Published:** 2022-07-08

**Authors:** Elisa Gamalero, Elisa Bona, Bernard R. Glick

**Affiliations:** 1 Dipartimento di Scienze e Innovazione Tecnologica, Università del Piemonte Orientale, 15121 Alessandria, Italy; 2 Dipartimento per lo Sviluppo Sostenibile e la Transizione Ecologica, 13100 Vercelli, Italy; elisa.bona@uniupo.it; 3 Center on Autoimmune and Allergic Diseases (CAAD), Università del Piemonte Orientale, 28100 Novara, Italy; 4 Department of Biology, University of Waterloo, Waterloo, ON N2L 3G1, Canada; glick@uwaterloo.ca

**Keywords:** sustainable agriculture, abiotic plant stress, plant growth-promoting bacteria, nitrogen fixation, plant-microbe interaction, plant microbiomes, soil bacteria

## Abstract

Many different experimental approaches have been applied to elaborate and study the beneficial interactions between soil bacteria and plants. Some of these methods focus on changes to the plant and others are directed towards assessing the physiology and biochemistry of the beneficial plant growth-promoting bacteria (PGPB). Here, we provide an overview of some of the current techniques that have been employed to study the interaction of plants with PGPB. These techniques include the study of plant microbiomes; the use of DNA genome sequencing to understand the genes encoded by PGPB; the use of transcriptomics, proteomics, and metabolomics to study PGPB and plant gene expression; genome editing of PGPB; encapsulation of PGPB inoculants prior to their use to treat plants; imaging of plants and PGPB; PGPB nitrogenase assays; and the use of specialized growth chambers for growing and monitoring bacterially treated plants.

## 1. Introduction

With a worldwide population of ~8 billion people that continues to grow, it is becoming increasingly difficult and expensive to feed all the people on our planet. Overall, about 3 billion people, a value that is destined to double by 2050, live in geographical regions such as South and East Asia, North Africa, and the Middle East that can be classified as drylands typically subjected to drought and salt stress. These environmental stresses which are mainly related to the increase in surface global temperature, represent a significant limitation for plant and crop production.

To increase the global food supply with only a limited amount of arable land, several approaches are possible. (1) We can expand the current use of chemical fertilizers and pesticides while trying to dramatically limit food wastage. (2) We can intensify the development, testing, and use of genetically engineered and genome-modified crops, a strategy that has been so far reasonably successful despite serious opposition to the genetic alteration of plants in many countries. (3) We can increase the deliberate use of plant growth-promoting bacteria (PGPB) and beneficial fungi such as mycorrhizae, and thereby slowly replace the use of chemicals in agriculture. Realistically, none of these approaches, by itself, is likely to be sufficient to increase agricultural productivity to the point where we can satisfy all the needs of the world’s peoples in the coming years. However, to increase the food supply and decrease the use of potentially deleterious chemicals (chemical fertilizers, herbicides, fungicides, and insecticides) in agriculture, decreasing food wastage, especially in the more developed world, plus, a combination of increasing the use of both genetic modification of crops and utilizing PGPB, currently appears to be a useful path to pursue. 

Most soils contain very high levels of bacteria (often estimated to be ~10^8^–10^9^ bacterial cells per gram of soil). Some of these bacteria are PGPB, some are phytopathogens and some do not have any discernible effect on plant growth. The knowledge of the physiological traits and mechanisms that are used by PGPB to promote plant growth has developed considerably over the past 20–30 years [1]. In fact, while only a small number of PGPB have been commercialized as adjuncts to agricultural practice, both the number of commercialized strains and the amount of farmland devoted to using this approach continue to increase with each succeeding year [1,2].

All land plants host a microbiome (defined as the pool of genes expressed by the microbial communities associated with plants) composed of bacteria, fungi, oomycetes, viruses, archaea, and protists [3,4]. These organisms primarily colonize the root environment, especially the rhizosphere (the soil portion immediately adjacent to the root), the rhizoplane (intended as the root surface), and, to a lesser extent the leaf surface (phyllosphere), seed (spermosphere), and internal (endosphere) plant environments [3]. Therefore, microbiomes associated with plants show a different degree of intimacy with the plant: while epiphytic microorganisms can live externally, endophytic microorganisms efficiently colonize the internal plant tissues. In this context, plants may be viewed as superorganisms that include their microbiomes, which provide several unique functions and features. However, notwithstanding an abundance of recent studies (reviewed in [5]), the knowledge on plant-associated microbiomes and their detailed effects on crop productivity, growth, health, and disease [6] is still scanty. Plants can take advantage of the rhizospheric microbiome by selectively inducing activities within microorganisms that support their development and survival [7]. In this way, rhizodeposition shapes the plant-associated microbiome, which in turn, can affect both plant metabolic pathways and exudate release. Based on this knowledge, Korenblum et al. [8] suggested the existence of crosstalk among the host–microbiome and metabolome, where plants perceive and understand the chemical communication among microbes and vice-versa. By considering the plants as an holobiont [9,10] communicating with the external environment, that in turn affects the interrelationships occurring in the holobiont itself, the authors [8] defined this circuit as a “metabolic circular economy” where molecules affecting rhizosphere interactions and plant health can be degraded, used, and modified by the members of the rhizosphere microbiome.

Using the term originally developed for microorganisms present in the human body, the microbial communities are indicated as plant microbiota [11]. A multitude of studies, recently reviewed by Glick and Gamalero, [5] have demonstrated that plant/root microbiota can play important positive roles in plant fitness and improvement of quality in different crop productions.

Until now, most of the knowledge and understanding of how PGPB promote plant growth has included studies of isolated bacteria examined in controlled laboratory conditions that have not considered the range of organisms and abiotic conditions that affect how these bacteria function in soil. In the soil, PGPB typically act together in groups or consortia, with the consortia being responsible for facilitating plant growth, that is, different bacteria within the consortia satisfy different plant needs. The attraction of both individual PGPB, as well as bacterial consortia of PGPB, to a particular plant is dictated, in part, by the range and concentration of small molecule root exudates produced by a particular plant [1]. Interestingly, even though the microbiota in most microbiomes contains both PGPB and deleterious microorganisms, for the most part, plants attract beneficial PGPB and not phytopathogens. Thus, despite the presence of phytopathogens in many microbiomes, the beneficial PGPB often protect plants from the deleterious effects of phytopathogens.

Diverse interactions, such as cooperation, inhibition, and competition can be established among the members of bacterial communities. Nevertheless, some members of the community may behave as neutral or avoid all interactions, resulting in community-intrinsic properties, or properties of bacteria that are shown only at the community level [12]. On the other hand, new members introduced into the community can induce a shift in the microbial community structure and, at the same time, be sensitive to the antagonistic molecules released by the original members [13]. Building synthetic communities (SCs) means scaling up the experimental evolution from one- or two-species interactions by combining the emergent members and molecular interactions occurring in a complex model system. Obviously, all the members of the SC must be able to grow on the laboratory media in order to allow the experimental procedure [14]. Significant increases in the efficacious use of PGPB or SC, together with concomitant decreases in the use of potentially harmful chemicals, require an increased understanding of how these beneficial bacteria function and how they interact with plants.

According to a recently proposed theory, plant adaptation to climate change is driven in the short term by the microbiome associated with the plant, while in the long term it will be directed by the eco-evolutionary interactions occurring between the microbiome and its plant host [15]. For this reason, it becomes more and more important to be ahead of this time and starting from now to develop new methods, techniques, and approaches for studying plant-microbe interactions. With this in mind, we wrote this review aiming to provide an in-depth overview of the methods currently available to study beneficial plant-microbes interactions, mainly at the root level.

## 2. Techniques to Study Plant-PGPB Interactions

### 2.1. Omics Techniques

#### 2.1.1. DNA Sequencing

Genomic studies of environmental samples have recently become one of the most efficient tools allowing one to get a large amount of knowledge regarding the evolutionary history, structural, functional, and ecological biodiversity [16]. The term “metagenomics” was first introduced in 1998 and was defined as “*the evaluation of all the genetic materials isolated directly from the relevant environmental samples*” [16]. It is the most commonly employed method in the study of the complex microbial community established in environmental samples through analysis of the nucleotide sequences [17]. Targeted or shotgun sequencing are the two main approaches used in metagenomic studies: the exploitation of one or the other approach largely depends on the type of environmental studies to be performed. Despite the approach used, it is true that conventional sequencing has allowed the construction of an avenue for the buildup of large barcode DNA reference libraries [16,18].

Next-generation sequencing (NGS) leads to the creation of a platform containing DNA sequence data extracted directly from environmental samples [19]. This large amount of data may have a number of applications such as the comparisons of microbiota present in diseased and healthy individuals [20]; biodiversity studies of the ecosystem [21]; and evolutionary studies of DNA [22]. The Illumina platform provides many millions of highly accurate reads and is currently preferred for metagenomic sequencing since it allows researchers to speed up the entire procedure at a more affordable cost [23,24].

#### 2.1.2. Whole PGPB Genomes

Whole Metagenome Shotgun sequencing includes DNA or RNA extraction from the community present in a specific environment, library construction, and short-read sequencing of the entire mixture of genomes or transcripts. Altogether, this leads to millions of short random DNA/cDNA fragments that can then be alternatively assembled or used as markers for specific groups of microorganisms or/and metabolic pathways. Although the Illumina platform is considered one of the most performant for meta-omic sequencing [23,24], other emerging platforms such as Ion Torrent and PacBio have been created. However, they have not yet reached the same spreading and utilization level compared to Illumina [25,26,27].

Whole-genome sequencing has been widely used in recent years to characterize several PGPB. This approach may be used to study the genomic basis of different phenotypic characteristics such as resistance to salt or heavy metals or plant growth promotion capability. For example, Zhang et al. [28] analyzed the whole genome of *Brevibacterium frigoritolerans* ZB201705 isolated from the rhizosphere of drought- and salt-stressed maize, revealing that this strain is able to synthesize many proteins known to be involved in the cell response to drought and salt stress. This suggests that *B. frigoritolerans* ZB201705 might be used as an inoculant in order to increase crop yield also under abiotic stresses. Moreover, Berrios [29] presented the genome of a PGPB, *Caulobacter segnis* CBR1, and contextualized its genomic features with the genomic features of sequenced *Caulobacter* strains demonstrating that the CBR1 genome harbors genomic features that are responsible for *Caulobacter* strains to enhance the growth and development of *Arabidopsis* plants. Duan et al. [30] elaborated the genome sequence of *Pseudomonas* sp. strain UW4 using pyrosequencing and closed the gaps between the contigs by directed PCR [30]. In this work, genes potentially involved in plant growth promotion such as those encoding for indole-3-acetic acid (IAA), ACC deaminase, trehalose, siderophore, acetoin, and phosphate solubilization were identified [30].

Whole-genome analysis has enabled “pan-genome” studies, which consist of a description of all the genes occurring in all the strains belonging to a species [31,32]. The pan-genome is composed of the core genome, defined as the genes universally shared by all strains belonging to a species, and the accessory genome, represented by genes detected only in some strains of the same species [33]. The concept of pan-genome applied to prokaryotes is widely accepted and the recent progress in this field aims to shed light on the mechanisms and process modulating the pan-genome structure itself [34]. Some of the largest pan-genome studies include pan-genomes of human opportunistic pathogens such as *Escherichia coli* and *Streptococcus pyogenes* [35,36]. These studies revealed that a negative relationship exists between the size of the pangenome and the number of genes comprising the core genome. In detail, pangenomes that are classified as “open” are larger in size, possess multiple genes acquired by horizontal gene transfer, and are characterized by a smaller fraction of core genes. On the other hand, “closed” pangenomes have a smaller size, a low number of genes derived by horizontal gene transfer, and a larger proportion of core genes [37]. It has been suggested that bacterial species with open pangenomes have a better environmental performance compared to bacterial species having closed pangenomes since they can colonize a wider variety of ecological niches and often have a dominant role in complex communities. The size of the core and accessory genomes is also strongly related to bacterial lifestyle. Those bacteria showing sympatric lifestyles live in strict contact with other organisms and create complex interactions with them, while allopatric bacterial species live in isolation. Therefore, bacterial species having sympatric lifestyles possess open pangenomes, while allopatric ones are characterized by closed pangenomes, having a lower amount of accessory genes [38].

As an example of a pangenome study applied to rhizosphere bacteria, Olanrewaju et al. [39], analyzed and compared the genomes of 10 PGPB belonging to the *Bacillus* genus, five strains were identified as *B. subtilis* and the other five as *B. velezensis*. Overall, the pangenome involved 777 core genes, with *Bacillus subtilis* strain BSA29 having the lowest amount of accessory and unique genes, and *Bacillus subtilis* R31 having the highest number of accessory and unique genes. Since the ratio between the core and the pangenome genes did not reach a plateau phase, the results obtained to date emphasize the fact that the pangenomes of these two species can be defined as open.

#### 2.1.3. Sequencing of Endophytic Genes

Numerous multifunctional agriculturally important microbes are found inhabiting internal plant tissues [7,40,41]. The role of endophytic microbes in agricultural biotechnology ranges from mitigating environmental stressors to improving plant growth and health [42]. The potential of endophytic bacteria to support plants growing under stressful conditions depends on several factors including the release of a multitude of bioactive and volatile compounds, such as phenolic compounds, exopolysaccharide, ethylene, auxins, organic acids, and siderophores [43], as well as the synthesis of the enzyme ACC deaminase, which prevent the ethylene level to reach inhibitory concentration for plant growth [44]. Microbe establishment inside the plant tissue typically occurs in five distinct steps: (i) molecular dialogue between the bacterial strain and the plant host consisting of the release of specific molecules in the root exudates, recognition of these compounds by the bacterial counterpart, and chemotactic answer towards the plant; (ii) adhesion to the root surface; (iii) biofilm formation; (iv) penetration of the root cortex and (v) colonization of the internal parts of a plant [45]. Each of these stages is mediated by various molecules driving dynamic expression changes occurring both in bacterial and host plant genes. To determine how these biomolecules exert these effects, more holistic strategies that employ multiple-omic approaches should be applied. Endophytic diversity culture-independent methods mostly depend on the total bacterial genomic DNA extraction from plant tissues. As the first step, the bacteria colonizing on the root surface must be detached [46] and this can be easily achieved using an aseptic peeling to remove the surface layers, or by vigorously shaking the plant tissues in a saline solution containing acid-washed glass beads, followed by washing in sterile distilled water. The bacterial genomic DNA is then extracted by homogenizing plant tissues [47]. The amplified gene fragments, representing the entire endophytic population living inside the plant tissues, can be analyzed using a plethora of available molecular DNA fingerprinting techniques such as Amplified rDNA Restriction Analysis (ARDRA), Denaturing Gradient Gel Electrophoresis (DGGE), Temperature Gradient Gel Electrophoresis (TGGE), or Terminal Restriction Fragment Length Polymorphism (T-RFLP). Alternatively, other techniques based on the analysis of the highly variable region between 16S and 23S rDNA (Automated Ribosomal Intergenic Spacer Analysis, ARISA) can be used to obtain the community fingerprinting. However, to date, all these DNA fingerprinting techniques have largely been overtaken by the development of metagenomics techniques applying NGS [46].

Starting with the pioneering work of Weilharter et al. [48], attention has been focused on the whole-genome sequencing of bacterial endophytes, more than on single genes. In this paper, the genome *B. phytofirmans* PsJN, isolated from onion roots colonized by the AM fungus *Glomus vesiculiferum* (now *Rhizoglomus vesiculiferum*), was characterized. The data revealed that the 8.2-Mb genome of strain PsJN consists of two chromosomes and one plasmid, together containing a total of 7405 genes, 73.7% of which had assigned functions. While a large amount of the coding sequences involved in essential functions, such as cell replication or central metabolism were located on chromosome 1, genes involved in accessory functions (i.e., tolerance to heavy metals) were on chromosome 2. Genes responsible for the synthesis of ACC deaminase (*acdS*-*acdR* operon) were found in the PsJN genome together with genes involved in two independent pathways of IAA production (indole-3-acetamide and the tryptophan side chain oxidase pathways). However, no genes involved in nitrogen-fixation or production of antibiotic molecules were detected.

Two years later, Mitter et al. [49] compared the whole genome sequence of the strain PsJN with the entire genome sequence of other eight bacterial endophytes (*Azospirillum* sp. B510, *Klebsiella pneumoniae* 342, *Methylobacterium populi* BJ001, *Pseudomonas putida* W619, *Pseudomonas stutzeri* A1501, *Enterobacter* sp. 638, *Azoarcus* sp. BH72, and *Gluconacetobacter diazotrophicus* Pa5) to assess the possible occurrence of common traits responsible for the endophytic lifestyle.

Efficient root colonization is the first requirement for successful endophytic behavior. Regarding this specific trait, four out of the nine bacterial endophytes *P. putida* W619, *Enterobacter* sp. 638, and *P. stutzeri* A1501, and *M. populi* BJ001, contained genes for curli fibers. Similarly, genes coding for agglutinins occurred in *P. putida* W619 and *Azoarcus* sp. BH72 and hemagglutinin genes (involved in eukaryotic cell colonization by bacteria) were detected in *B. phytofirmans* PsJN, *K. pneumoniae* 342, *P. putida* W619, and *Enterobacter* sp. 638. Despite their well-known ability to colonize the roots of different crops, in *Azospirillum* sp. B510 and *G. diazotrophicus*, PAl5 genes responsible for cell adhesion were not detected [49].

Interestingly, strain PsJN carries two quorum sensing operons, as well as genes encoding cellulases and endoglucanases. Finally, genes involved in the synthesis of flagellin (ensuring plant internal colonization), pollutant degradation, tolerance to heavy metals, and a variety of enzymes used to modulate oxidative stress were all found. Based on previous work some features such as motility and chemotaxis have been suggested to be necessary for the endophytic lifestyle. However, the genome of strain *Klebsiella pneumoniae* 342 did not show any genes involved in flagella synthesis. On the other hand, genes responsible for ROS detoxification and for the synthesis of N-acylhomoserine lactones (NAHLs) are common to the genomes of the nine bacterial endophytes considered [49].

Regarding the possible adaptation to fluctuating environmental conditions, among the nine bacterial endophytes, only *G. diazotrophicus* PAl5 and *M. populi* BJ001 carried a high number of genetic mobile elements (109 and 72, respectively), suggesting that the other seven bacterial species exploit mechanisms other than horizontal gene transfer to adapt to rapidly changing conditions. Moreover, the nine strains differed in their iron uptake capability through siderophores; genes involved in siderophore production were found only in four out of the nine bacterial endophytes (*B. phytofirmans* PsJN, *K. pneumoniae* 342, *Enterobacter* sp. 638 and *P. stutzeri* A1501). However, the other five bacterial isolates showed genes encoding outer membrane iron receptors, possibly involved in the capture of ferri-siderophores synthesized by other soil microorganisms [49]. Whole-genome analysis revealed that all the bacterial endophytes, except for *P. stutzeri* A1501 have quorum sensing-related genes. *B. phytofirmans* PsJN has two operons LuxI-LuxR on chromosome 2 leading to the synthesis of four NAHLs, one of them (3-hydroxy-C8-homoserine lactone) involved in swimming motility and in rhizosphere colonization in *Arabidopsis thaliana* [50]. As previously suggested by Horswill et al. [51] the occurrence of multiple signal molecules in the cell-to-cell communication network may stabilize the intra- or inter-species bacterial communication in an environment affected by environmental perturbations.

The release of proteins plays a central role in plant–microbe interactions. Two secretion systems have been described in bacteria: type I, type III, type IV, and type VI translocate proteins directly across the internal and external membranes, while type II and type V secretion systems T2SS, T5SS transport proteins first to the periplasm and then to the outer membrane. All these protein transport systems were found in the genomes of the nine bacterial endophytes. In particular, T1SS occurred in all the strains except for *B. phytofirmans* PsJN and T2SS in six strains except for *K. pneumoniae* 342 and *Azospirillum* sp. B510. Interestingly, in the genome of *B. phytofirmans* PsJN genes coding for at least four different secretion systems (T2SS, T3SS, T4SS, and T6SS) were detected, more than the other considered endophytic strains [49].

The overall information indicates that some of the considered genetic traits are common to the nine bacterial endophytes. However, it appears that the different bacterial species utilize somewhat different strategies to colonize internal plant tissues. In fact, strains *Azospirillum* sp. B510, *G. diazotrophicus* PAI5, *Azoarcus* sp. BH72, and *K. pneumoniae* 342 are typically associated with grasses where they release fixed nitrogen, while *M. populi* BJ001, *P. putida* W619, and *Enterobacter* sp. 638 are unable to fix nitrogen and live in poplar trees. On the other hand, *B. phytofirmans* PsJN can efficiently be established both in the rhizosphere and inside the tissues of a variety of unrelated plant species. It is thus reasonable to hypothesize that individual bacterial endophyte genomes have evolved following the specific requirements of their plant hosts [49].

Shortly thereafter, Ali et al. [52] published a similar study in which nine endophytes including *Burkholderia phytofirmans* PsJN, *Burkholderia* spp. strain JK006, *Azospirillum lipoferum* 4B, *Enterobacter cloacae* ENHKU01, *Klebsiella pneumoniae* 342, *Pseudomonas putida* W619, *Enterobacter* spp. 638, *Azoarcus* spp. BH72, and *Serratia proteamaculans* 568 were studied to determine which genes appeared to be involved in endophytic behavior. This study concluded that there was a set of 40 genes that were largely conserved between these strains and that these 40 genes were likely involved in specifying endophytic behavior.

Since then, efforts have been made to sequence complete endophytic bacterial genomes belonging to *Azospirillum*, *Arthrobacter*, *Bacillus*, *Burkholderia*, *Devosia*, *Dyadobacter*, *Enterobacter*, *Gluconacetobacter*, *Leifsonia*, *Methylobacterium*, *Microbacterium*, *Micrococcus*, *Paenibacillus*, *Pantoea*, *Phyllobacterium*, *Pseudomonas*, *Rhanella*, *Rhodanobacter*, *Rheinheimera*, *Sphingomonas*, *Stenotrophomonas*, *Pedobacter*, *Pseudoxanthomonas*, and *Variovorax* genera, isolated from various plants in order to better understand the potential beneficial effects that these strains can provide to the host plant (for a recent review see [53]).

#### 2.1.4. Antibiotic Resistance Genes

According to the World Health Organization (WHO), antibiotic resistance is defined as “*an increase in the minimum inhibitory concentration of a compound for a previously sensitive strain*” [54]. However, in this definition, the concepts of intrinsic resistance [55] as well as of resistance genes naturally occurring in the environment [56] are neglected. The results obtained in different environmental microbiology works show that antibiotic resistance genes have been found not only in diverse environmental samples, such as soil [57] or oceanic cold-seep sediments [58] but also in pristine environments predating the antibiotic era [59,60]. More recently, Nesme et al. [61] revealed that genes with significant similarities to known antibiotic resistance genes, occur in all environments, clearly indicating the spreading of antibiotic resistance genes in environmental samples [61]. Soil is recognized as the most prominent reservoir harboring as much as 30% of all different known resistance genes found in sequence databases. This information supports the hypothesis of the existence of an abundant environmental (especially in soil) reservoir and highlights the relevance of antibiotic resistance genes for bacterial ecophysiology at the ecosystem level. Altogether, these data indicate that genes of antibiotic resistance or antibiotic synthesis should be considered essential for the survival of many environmental bacteria, whatever their intrinsic function may be and even though the specific selective forces driving their dissemination is still unknown.

The principle of metagenomics is to apply standard molecular techniques based on DNA extracted directly from the environmental sample. Since the development of metagenomics has been combined with next-generation sequencing, many previous critical technical limitations have been alleviated [62]. The complex antibiotic resistome (including every antibiotic resistance gene) ecology can be deciphered only by considering its environmental aspect (i.e., outside hospitals’ walls).

Understanding the specific features of a complex environment such as the soil is a critical point when we aim to define all the interactions being established among all the components of this biota, from cells to molecules. In this context, soil is an incredibly rich environment if considering microbial abundance and species diversity. In fact, as previously mentioned, 1 g of soil may contain 10^6^–10^9^ bacterial cells of 10^3^–10^6^ different bacterial species [63].

Environmental factors (e.g., particle size, pH, water availability, vegetation cover, etc.) are related to the distribution of bacterial and/or fungi species diversity in soil [64,65]. For sure, in soil, competition and antagonism mediated by the synthesis and release of antibiotic molecules frequently occur and this can, at least partly, explain why antibiotic producers and resistant strains become dominant in soil. In fact, soil is considered an open and connected ecosystem characterized by constant and intricate interactions among all the biosphere compartments, leading to a high frequency of genetic material exchange also among ecologically distinct taxa usually found in other ecosystems.

### 2.2. Definitions of Metagenomic, Metaproteomic, Metatranscriptomic, and Metabolomic

Metagenomic, metatranscriptomic, and other whole community functional assays, as suggested by Segata and coworkers [66], provide instruments to study complex ecosystems involving host organisms, biogeochemical environments (interactions occurring in soil/rhizosphere/plants), pathogens, biochemistry and metabolism, and the interactions among them. All the information produced by -omic techniques provides the tools to answer important biological questions in microbial community biology. According to the synecological view, microbial communities can be considered as complex biological entities interacting with the environment, host organisms, as well as transient microbes. Although several studies aimed to provide key insights, the availability of predictive models studying the interactions within these ecosystems is currently limited [66]. Some of the issues addressed by these studies include the longitudinal variation of these systems which may be due to multispecies metabolism or the characterization of microbe-microbe interactions and/or community’s co-evolution due to ecological pressure.

The main objective of meta-omic analysis is to identify a set of microbial organisms, genes, variants, metabolic pathways, or ecological functions that, taken together, can characterize the microbial community living in an environmental sample [66]. Metatranscriptomics (c-DNA sequencing) combined with metaproteomics and community metabolomics complement the description of the whole community obtained by metagenomics [67,68]. Metagenomic and metatranscriptomic approaches provide a full description of the genomic composition and diversity within and across communities through molecular culture-independent sequencing methods, including targeted sequencing of 16S rDNA in bacteria, rDNA 18S in eukaryotes, and ITS (Internal Transcribed Spacer in fungi) [69], as well as whole-metagenome shotgun (WMS) sequencing. Multi-omics data, resulting from the combination of metagenomic, metatranscriptomic, metaproteomic, and metabolomic data have been implemented in several studies in order to reach a better knowledge of the soil microbiome and on the molecular changes taking place at the community level induced by environmental perturbations [70].

**Figure 1:**
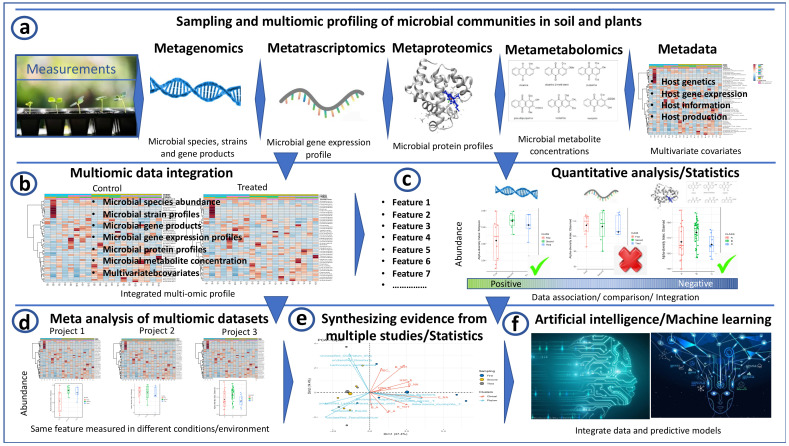
The figure shows the role and the interactions between the multi-omic approaches useful for characterizing microbial communities in soil and in the rhizosphere. Each panel shows a different possible workflow. Panel (**a**) defined the initial approach, or the possible approaches used to study the different aspects of microbial communities. The initial analysis determines the type of data that will be inserted into the workflow (panel (**b**)). Based on this choice, the statistical approaches (panels (**c**,**d**)) are useful to identify the differences between the considered microbial communities that can change. Finally, panels (**e**,**f**) suggest the integration of these different kinds of data while also using a machine learning approach in order to produce a predictive model.

Table 1 reported the main advantages and disadvantages of all the -omic techniques when considered alone; the way all these techniques can be combined is illustrated in Figure 1.

#### 2.2.1. Transcriptomics

The main goal of transcriptomic analysis of plant-associated bacteria using RNA sequencing (RNA-seq) or gene expression microarray approaches, is to reveal genes that are differentially expressed under specific environmental conditions. Most of the current scientific works exploiting transcriptomic to study plant-microbe interactions have been performed by culturing bacteria separately from the host plant. RNA-seq was used, for example, to detect genes responding to the presence of a plant extract [71,72]. However, by using the metatranscriptomic approach, sequencing the transcripts of the whole community directly from the environmental samples, more information on the simultaneous transcriptional state of a plethora of microorganisms can be obtained.

One study of plant-bacterial interaction by transcriptomic analysis includes the work of Stearns et al. [73], which elaborated on some of the changes that occur in canola plants treated with the PGPB *Pseudomonas* sp. UW4. That study found that transcription of genes encoding plant hormone regulation, secondary metabolism, and stress response were upregulated in plants treated with the PGPB. On the other hand, upregulation of genes for auxin response factors and downregulation of stress response genes was detected only when canola was inoculated with the wild-type strain able to synthesize ACC deaminase and not in plants inoculated with the ACC deaminase minus mutant of the PGPB. Importantly, this work led to a new model of plant growth promotion involving both ACC deaminase and auxin signaling.

The efficient and stable establishment of PGPB in the rhizosphere depends on many molecular and cellular factors, such as the capability to move in response to different environmental stimuli, the metabolic versatility, the ability to form biofilm, and the release of secondary compounds involved in the dialogue between bacterial cells and the host plant. RNA-seq transcriptomic analysis has been used to study the mechanisms at the base of the interactions of the PGPB *Delftia acidovorans* RAY209 (commercially available as BioBoost Liquid; Lallemand Plant Care) with canola and soybean plant roots, with special attention given to the colonization process. Following gene expression after two (early colonization) and seven days (sustained colonization) from adding the inoculum to canola or soybean plantlets grown hydroponically, it has been demonstrated that *D. acidovorans RAY209* showed both a core regulatory and a plant host-specific regulatory response to root colonization. A high number of significantly differentially expressed genes by strain RAY209 were detected compared to bacterial cells suspended in the medium to root-attached cells during early colonization on soybean (823 genes). Conversely, 847 genes were differentially expressed by bacterial cells suspended in the medium compared to root-attached cells during sustained colonization of canola roots. However, once cells are firmly attached to the roots of the two plant species, a high level of a fasciclin gene homolog encoding a protein mediating adhesion, genes encoding hydrolases, and genes involved in multiple biosynthetic processes as well as in membrane transport were found. Interestingly, during early attachment to the roots of canola or soybean, transcription of ABC transporter occurs in the PGPB strain, while other transporter genes were expressed only in association with each plant species. These data indicate that RAY209 can specifically adjust its cellular activities to adapt to the plant species considered [74].

Very recently, the effects of the biocontrol agent *Bacillus subtilis* MBI600, commercialized by BASF, on the transcriptome and metabolome levels of cucumber roots were investigated [75]. An analysis of the differentially expressed genes was performed on cucumber roots 24 and 48 h after the inoculum was added and compared to the genes expressed in untreated plant roots. The expression of genes involved in signaling (transcription factors with ethylene response and LRR proteins), defense against phytopathogens (peroxidase, endo-1, 3(4)-beta-glucanase, pathogenesis-related protein PR-4 and thaumatin-like protein) and plant growth (potassium channel SKOR, potassium transporter 5, and zinc finger protein GIS4, indole-3-acetic acid-induced protein ARG7, and auxin-responsive proteins) were induced after 24 h after the plant inoculation with the biocontrol agent. According to the KEGG pathway analysis, the differentially expressed genes belonging to biocontrol-related pathways were related to plant immunity and represented by MAPK (Mitogen-activated protein kinase) signaling.

Finally, metatranscriptomics has been applied to identify differentially expressed bacterial genes during *Arabidopsis* growth [76] and invasion by a fungal pathogen [77].

#### 2.2.2. Proteomics

Proteomics and metaproteomics approaches are typically based on liquid chromatography-tandem mass spectrometry technology and allow one to obtain semi-quantitative information on the diversity of bacterial proteins synthesized in a specific environmental sample. Soil metaproteomics uses proteins to understand how microbes contribute to soil ecosystem changes, giving information regarding secreted enzymes in soil and the microbial species that exert these metabolic capabilities. These techniques are based on several steps: more in detail, after sample collection protein extraction is performed, it is followed by isolation and fractionation, mass spectroscopy analysis, and finally, the comparison with a proteome database [72].

An interesting example of using proteomics to better understand plant-bacterial interaction can be found in the study by Cheng et al. [78]. In that study, 72 PGPB proteins with significantly altered expression levels were identified in the presence of canola root exudates. Most of these proteins were involved in nutrient transport and utilization, cell envelope synthesis, and transcriptional or translational regulation. The expressions of four of these proteins that showed large changes in expression in response to canola root exudates were examined in detail, and three of them were shown to significantly affect plant-bacterial interactions.

Metaproteomics has been used to characterize the phyllosphere metaproteome of forest trees [79], to detect proteins differentially secreted by PGPB strains in response to root exudates [80], and to identify both the organisms and proteins responsible for nitrogen fixation and methane oxidation in rice fields [72,81]. In a recent study [82], a metaproteome approach was applied to produce a wider view of the active members of the bacterial community in a vineyard subjected to integrated pest management, whose bacteriome was previously characterized by metabarcoding [83]. By comparing the data from these two studies, it became evident that the dominant members of the bacterial community in both the bulk soil and grapevine rhizosphere did not precisely overlap with the active members of the bacterial community. The combination of metabarcoding and metaproteome techniques to the same environmental samples allowed for two fundamental questions in microbial ecology to be addressed, that is, “who?” and “what?”, thus obtaining a complete description of the bacterial actors and their roles in the vineyard environment.

#### 2.2.3. Metabolomics

Targeted or untargeted metabolomics can be used to measure changes in specific metabolite levels in response to a given treatment [72]. In fact, different bacterial elements, such as the nodulation (Nod) genes encoding for the Nod factors, directly affect the host plant or microbial metabolism [72]. Mass spectrometry (MS)-based metabolomic, and lipidomic measurements typically require a specific extraction process for each kind of molecule to be MS-compatible [84]. These challenges have favored the development of methods allowing simultaneous metabolite and lipid extraction, based on the use of smaller sample volumes or masses, improving the accuracy, and providing faster sample preparations for all analyses [85].

Another approach to studying metabolites that are present in soil is nuclear magnetic resonance (NMR) metabolomics, a tool that can be used to analyze the set of responses of an organism to different environmental stimuli and can reveal pre-symptomatic signs of stress and disease [86]. This approach could be used both for targeted and fingerprint metabolomics as potential indicators for soil health. For example, Rochfort and coworkers [87] employed proton nuclear magnetic resonance spectroscopy (^1^H NMR) for the metametabolomic analysis of natural and agricultural soils. The metabolomic methodologies employed in this work were based on grinding and extraction with sonication thus enabling the characterization of both extracellular and intracellular components of soil [87].

Compared to metagenomics, both the cost of the equipment and the technical level of expertise necessary to perform metabolomic analysis are higher and make this approach less accessible than DNA sequencing. Moreover, the sizes of public databases containing metabolite references are limited, so it can quite often be difficult to correctly assign a detected metabolite to a specific organism. However, metabolomics represents a powerful tool to detect and quantify small molecules and identify specific metabolic pathways involved in plant–microbe interactions thus obtaining a comprehensive view of the complexity of interrelationship occurring among the members of the microbiota and between the microbiota and its host plant [72,88].

A metabolomic approach was used to describe the modulation of rhizosphere community assembly and succession according to the molecules released by root exudation, especially aromatic organic acids, from grass (*Avena barbata*) [89]. More recently, the metabolome of *Oryza sativa* cv. Nipponbare was analyzed following the inoculation with 11 PGPB strains (eight of them belonging to the genus *Azospirillum*, and the other three represented by *Herbaspirillum seropedicae* SmR1, *Paraburkholderia phytofirmans* PsJN, *Burkholderia glumae* AU6208) using UHPLC-DAD-QTOF (UltraHigh-Performance Liquid Chromatography-Diode Array Detector-Quadrupole Time-of-Flight). The subsequent statistical analysis revealed the occurrence of a metabolic signature common to all of the PGPB inoculated plants, with a low level of three alkylresorcinols and a high amount of three hydroxycinnamic acid (HCA) derivatives (feruloylquinic acid, N-p-coumaroylputrescine and N-feruloylputrescine). Since hydroxycinnamic acid-based molecules are known to modulate plant defense, these workers decided to inoculate plants with the rice pathogen *Burkholderia glumae* AU6208, to study its impact on the synthesis of HCA derivatives. The results obtained highlighted the fact that plant inoculation with a pathogen, differently from what was observed after PGPB inoculation, leads to a lower accumulation of HCA derivatives suggesting the ability of the plant to perceive the colonization by a plant beneficial bacterium versus a plant deleterious one through the up or down accumulation of these HCA molecules [90].

### 2.3. Imaging

#### 2.3.1. Labeling Bacteria

Once PGPB are released into the environment as biofertilizers or biocontrol agents, it is important to monitor their survival, behavior, and activity in the soil. Usually, the first prerequisite in tracking bacteria in complex environments is to distinguish the introduced strain from the autochthonous microbiota. Therefore, markers delineating the introduced strains should be specific and stable even under open field conditions [91,92]. Several serological and molecular markers have been developed during the 1980–1990 decade.

One of the first and most widely used markers is antibiotic resistance especially to rifampicin, kanamycin, and streptomycin, based on the selection of spontaneous chromosomal antibiotic-resistant mutants or following Tn*5* mutagenesis. Once the PGPB strain is tagged with antibiotic resistance, its density in soil or in the rhizosphere can be easily evaluated by colony counting on selective media that includes the presence of a specific antibiotic. Obviously, with this method, only the culturable fraction of the strain is monitored. However, it should be considered that in nature strains tagged with antibiotic resistance can pose serious concerns regarding the possible spread of antibiotic resistance genes in the environment due to horizontal gene transfer. Consequently, the deliberate release of PGPB tagged with antibiotic resistance genes is not recommended [93,94].

The use of a fluorochrome to visualize through microscopy, by counting bacterial cells, or through flow cytometry is simple, inexpensive, and well-established. Moreover, by coupling a fluorochrome having levels of different membrane permeability (i.e., propidium iodide staining cells with damaged membranes and Sybr green staining all living cells), it is possible to discriminate between living and dead cells. For example, a procedure for the optimization of staining protocols for environmental samples, based on these two fluorochromes has been developed for flow cytometry enumeration of bacterial cells in environmental samples [95]. However, the use of fluorochromes does not allow researchers to distinguish the introduced bacterial strain from the indigenous microflora and thus, this technique is of limited use.

The above-mentioned limitation can be overcome by the exploitation of strain-specific antibodies directed against the introduced bacterial strain (usually raised against cell membrane proteins), conjugated with fluorochrome, enzymes, or radioisotopes. Several manuscripts have reported studies of the root colonization dynamics by labeled PGPB using immunofluorescence [96,97,98,99], offering both a qualitative and quantitative characterization of this phenomenon. Here, antibody specificity is the main factor to take into consideration.

**Figure 2:**
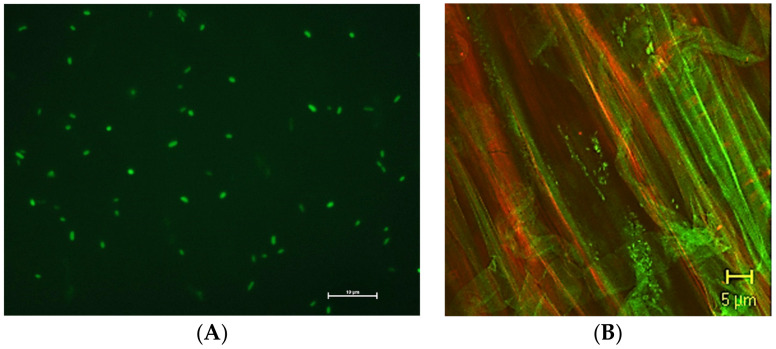
(**A**) A bacterial suspension of GFP-tagged *Pseuodomonas migulae* 8R6 cells. Epifluorescence microscope, 100X objective lens. Photo courtesy of Patrizia Cesaro. (**B**) Bacterial cells of *P. fluorescens* 92GFPrk along the primary root of a 7-day-old tomato plantlet. Confocal Laser Scanning microscope, 100X objective lens.

Subsequently, a great deal of interest was generated by the use of molecular marker genes such as the one encoding green fluorescent protein (GFP) (Figure 2) isolated from the jellyfish *Aequorea victoria* [100].

Since the isolation of the *gfp* gene, several other fluorescent proteins including enhanced GFP (EGFP), enhanced cyan fluorescent protein (ECFP), and enhanced yellow fluorescent protein (YFP) have been developed [101]. Although a red derivative of GFP was impossible to obtain, this issue was bypassed by the discovery of DsRed isolated from the coral *Discosoma striata* [102]. The availability of many proteins with different excitation and emission wavelengths, allowed the coupling of autofluorescent proteins and the visualization of several different metabolic processes in a single cell or in different cells in a complex system using fluorescent and confocal laser scanning microscopy [101]. The information obtained by microscopic observation should be complemented by digital image analysis with specific software able to provide data on cell count, shape, morphology, surface area, and volume. This kind of analysis consists of image acquisition through a digital system, the elimination of the background noise originating from the matrix (background can be very intense in soil, roots, and leaves), and the identification of the bacterial cells to be analyzed and their characterization [103].

Bacterial cells expressing GFP are easy to obtain by transconjugation [104,105] and commercial kits are available to accomplish this task. GFP can be constitutively expressed, is stable in aerobic environments, generally does not affect the fitness of the host, and is not modified by the metabolic activities of the bacterial host cells [106]. GFP expressing cells can be visualized and counted by flow cytometry. For example, by coupling dilution plating, microscopic observation, and flow cytometry, the amount of culturable, viable but not culturable (VBNC), and total cells of *P. fluorescens* 92rkG5 was determined along the primary root of tomato plants grown under controlled conditions [107]. However, GFP markers are not well suited for studying bacterial biofilms where oxygen depletion occurs due to bacterial metabolism. For this kind of application, it has been proposed to use the small tag named FAST (Fluorescence-Activating and Absorption-Shifting Tag), which becomes fluorescent after the addition of an exogenous fluorescent dye thereby avoiding the sensitivity to oxygen starvation [108].

**Figure 3:**
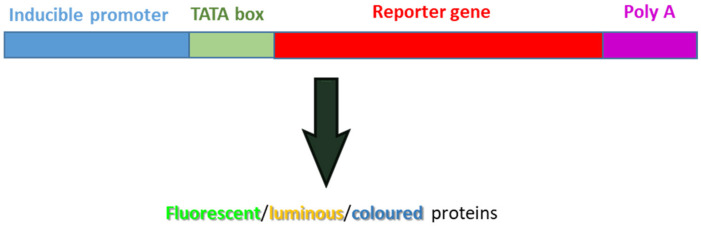
Typical construction of a reporter system. The reporter gene is usually under the control of an inducible promoter. As a result of the gene expression, the transformed cells appear fluorescent, luminescent, or colored when performing a specific activity.

When studying rhizosphere ecology, the idea of visualizing bacterial cells at the exact instant that they are expressing a specific metabolic activity is very attractive. To realize this aim, several reporter genes have been developed [109]. By definition, a reporter gene is a gene that can be artificially introduced before a gene of interest in a bacterial chromosome or plasmid thus conferring onto the bacterial cells the capability to be easily identified and visualized [110] (Figure 3). GFP and derivatives (under the control of an inducible promoter), as well as luminescence (*lux*AB and *luc*) and chromogenic (*gus*A and *lac*Z) genes, are the most commonly used genes in environmental ecology.

The discovery of the *lux* operon from the marine bacterium *Vibrio fisheri* and the knowledge of its regulation led to the widespread employment of the luciferase system as a reporter gene. Similarly, the luciferase *luc* genes isolated from the firefly *Photinus pyralis* [111] and the luminous click beetle *Pyrophorus plagiopthalamus* [112] can be easily expressed in bacterial cells (for a review, see [92]). For example, a single copy of a *mer-luc* marker gene cassette was introduced into the chromosome of a *Pseudomonas fluorescens* strain isolated from a birch tree (*Betula pendula*) rhizosphere. The survival of the strain in forest soil exposed to different temperatures was assessed, in a microcosm system, by evaluating the luminescence due to the *luc* gene, and the number of light-producing colonies on a medium containing HgCl_2_ and by PCR amplification of the *luc* gene [113].

Bacteria expressing the *E. coli* GUS gene encoding beta-glucuronidase appear blue when cultivated on a medium containing the substrate analog X-gal (5-bromo-4-chloro-3-indolyl-β-d-galactopyranoside) and the inducer IPTG (isopropyl β-D-1-thiogalactopyranoside).

Similarly, the *lacZ* gene encoding beta galactosidase in *E. coli* mediates the conversion of the colorless substrate 5-bromo-4-chloro-3-indolyl-3-o-galactoside to a deep blue colored product, which has been used as a reporter gene in diverse organisms.

Another fundamental issue when studying microbial ecology is the evaluation of living and active bacterial cells [114]. Traditional fluorescence in situ hybridization (FISH) [115] is based on the use of probes targeting the 16S ribosomal RNA (rRNA) allowing the visualization of active cells by fluorescent or confocal microscopy. A typical protocol consists of a cell fixation step (after which the cells lose their viability), a permeabilization phase to facilitate the internalization of the fluorescent probe and its combination with the target, and finally, a hybridization step. This popular technique, which uses probes designed for a specific taxonomic group, allows the visualization of active bacterial cells belonging to complex communities. Since the initial development of this technique, several modifications of the original protocol have been proposed. Recently, fluorescent in situ hybridization of transcript-annealing molecular beacons (FISH-TAMB) has been developed, characterized by the omission of the fixation step, thus working with living cells. This technique is reliable in the labeling of intracellular mRNA targets, differentiating among diverse transcriptional states, detecting both active and rare taxa, and maintaining cell viability [116]. Moreover, one of the main drawbacks of the original FISH protocol is the fact the technique can simultaneously detect only a few taxonomically different bacteria. A multicolor FISH approach proposed by Lukumbuzya et al. [117], based on the use of probes tagged with eight fluorochromes with different excitation and emission ranges, has been demonstrated to be suitable for the characterization of complex microbial communities in several environmental samples.

The combination of FISH with other techniques, such as microautoradiography (MAR–FISH), Raman microspectroscopy (Raman–FISH), and Secondary Ion Mass Spectrometry (FISH-SIMS) has been applied to environmental samples to visualize, identify, and quantify the incorporation of labeled substrates by individual microorganisms in complex microbial communities [118].

Many other techniques have recently been developed and tested to visualize and count environmental bacteria and to determine their viability and activity. For a more complete overview, see [114].

#### 2.3.2. WinRhizo System

Soil is a complex system where its chemical, physical, and biological properties within a given area are highly heterogeneous [119]. In this environment, plant root development in any space is a measure of the plant’s ability to exploit the unevenly distributed nutritional resources. Plant root development is strongly affected by the establishment of symbioses (with rhizobia or mycorrhizal fungi or both), the microorganisms living both on the surface (epiphytes) and the inside (endophytes) of the root, by the release of a plethora of different compounds by the soil microflora, by the availability of macro and micronutrients, and by diverse environmental parameters such as pH, water availability, oxygen gradient, and soil temperature.

Altogether, these variables determine how the root system of any particular plant grows and develops. There are at least four plant biology branches studying the shape of the root systems [120]. Root morphology analyzes “*the surface features of a single root axis as an organ*, *including characteristics of the epidermis such as root hairs*, *root diameter*, *the root cap*, *the pattern of appearance of daughter roots*, *undulations of the root axis*, *and cortical senescence*”. Root topology studies “*how individual root axes are connected to each other through branching*”. Root distribution refers “*to the presence of roots in a positional gradient or grid*”, which is related to the root biomass or root length as a function of factors such as depth in the soil, distance from the stem, and position between neighboring plants. Finally, root architecture looks at the “*spatial configuration of the root system*” [120]. Root architecture is a descriptor of multiple axes and, for this reason, once we get information on root architecture, we also know topology and distribution.

**Figure 4:**
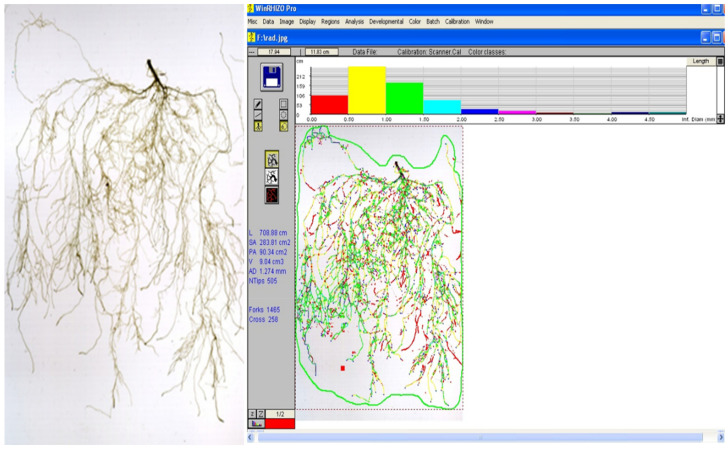
Scanned image of a tomato root system (**left**) and the corresponding WinRhizo analysis (**right**) showing the measurement of the total root length, surface area, projected area, volume, mean diameter, and tip number. In the upper part of the image, the root parameter classification (in this case root diameter) according to a 0.5 mm increase is reported.

Since the efficacy of nutrient and water uptake and transport, and consequently plant productivity, is strongly related to root development, the analysis of root architecture has become an important predictor of the yield and health status of a plant. To easily analyze root traits while avoiding human mistakes, several image analysis software programs have been developed, with WinRHIZO™ (Regent Instruments Inc., Quebec City, QC, Canada) being one of the best known. WinRHIZO™ is composed of an acquisition system of the image. This system includes a special high-quality scanner and lighting system with software that can readily convert root images into data on root morphology including total root length, area, volume, diameter, and branching; topology; architecture; and color analyses (Figure 4). The same instrument and computer software can also be used to calculate data for leaves such as the total projected area [121]. Obviously, this system represents a major step forward compared to older and much less accurate manual methods, however, the cost of this system may be not affordable for every laboratory. In this analysis, the washing and handling of the root must be performed very gently to avoid breaking the finer side roots and at the same time eliminate the attached substrate (soil, sand, peat, etc.). In addition, the roots must be carefully and thoroughly opened to ensure a good quality analysis limiting, as much as possible, the overlap between different root segments. Notwithstanding the very detailed information that can be obtained from this sort of analysis, this procedure can be difficult, painstaking, and time-consuming.

This system is currently used to study root development in different plant species and especially the root modification induced by Arbuscular Mycorrhizal Fungi (AMF) and PGPB [122,123,124,125,126,127]. However, it is important to remember that one of the first papers published on the modification of root morphology induced by AM fungi was published prior to the development of this technology and was entirely based on manual measurements [128].

Besides measuring root developmental traits, it has been proposed to use the analytical function color analysis of WinRHIZO™ coupled with 2,3,5-triphenyltetrazolium chloride (TTC) staining to evaluate metabolically inactive and active parts of plant roots. Thus, for example, while active clover (*Trifolium repens* L.) roots appeared dark red, bright red, or pale red, inactive ones were unstained [129]. More recently, the same function has been exploited to evaluate the arbuscular mycorrhizal colonization rate (the ratio of the fungal body to the plant root area) in a micrograph of seedlings of *Chengiopanax sciadophylloide*s, a woody species able to accumulate radioactive cesium sampled in the Yamakiya District of Fukushima, Japan several years after the 2011 nuclear disaster [130].

#### 2.3.3. Other Imaging Systems

As mentioned previously, the WinRHIZO™ System is costly and this represents a significant barrier to its use, especially in poorer countries. Therefore, scientists have developed other image analysis software packages, and some of these are available for free. One of these is ImageJ (formerly, NIHImage), an image processing software package developed at the National Institutes of Health of the United States [131]. This approach is based on the transformation of the considered image into a binary image, which is further analyzed using a threshold level that needs to be established manually. Unfortunately, this software is not user-friendly. To overcome this issue, Tajima and Kato [132] compared the results obtained using 16 different algorithms available in the ImageJ package for image processing with those obtained by the WinRHIZO™ system. Among them, the Triangle algorithm was identified as the best binary conversion method, although it overestimated the root length compared with the data provided by the WinRHIZO™ system. Fortunately, it was determined that similar values for the two systems could be obtained by multiplying the data obtained through Image J by 2/3. Subsequently, a macro of Image J, i.e., Image J Rhizo, has been developed as an open-source application, thus providing a new accurate tool for root image analysis to researchers who cannot afford the cost of commercial software packages. The efficiency of Image J Rhizo has been compared to that of WinRHIZO™, and the data confirmed that the values obtained measuring total root length were linearly correlated [133].

Similarly, Delory et al. [134] compared the root length data obtained by WinRHIZO™, Image J Rhizo RHIZO, and the manual line intersect method proposed by Tennant [135]. Overall, the results highlighted the fact that it is necessary to carefully interpret the data acquired through different methods. These authors recommended using a stain to enhance the contrast between finer roots and the background and to avoid as much as possible roots overlapping during the analysis.

Another root image analysis system, called RhizoVision Explorer (https://www.rhizovision.com/ accessed on 22 March 2022), has been recently developed and made available as open-source software characterized by an easy-to-use interface, fast image processing, and reliable measurements. RhizoVision Explorer was successfully validated by comparison with WinRHIZO™ showing a good overlapping of the results of the two approaches on the analyzed parameters except for root volume which was drastically underestimated by WinRHIZO™ [136].

Image analysis has received considerable attention since its possible application in field conditions in the context of precision agriculture. Precision agriculture has been defined as the scientific efforts aimed at enhancing crop yields and assisting management decisions using high technology sensors and analysis tools. These types of systems have been recently applied in the detection of soil-borne diseases due to phytopathogenic microorganisms in plants. As an example, a commercial optical sensor was used for the detection of Flavescence Dorée and Esca Disease in grapevine (*Vitis vinifera* L. cv. Dolcetto). Flavescence Dorèe is a disease caused by a phytoplasma carried by the insect vector *Scaphoideus titanus* and is characterized by plant growth inhibition, reddening of leaves starting from veins and rolling downward. Esca Disease is a fungal disease, whose main symptoms are foliar inter-venal necrosis and chlorosis. Each of these diseases can cause a significant reduction in plant yield. Usually, the detection of infected plants in a vineyard is based on the observation of symptoms performed by an expert plant pathologist followed by molecular analysis to confirm the diagnosis. With the support of commercial optical sensors (OptRx^®^) mounted on a transport system (often a quadbike, but drones are also used) and connected to a mobile PC equipped with GIS and RTK-GNSS software, the health status of grapevines in three vineyards was monitored. This optical sensor measures reflectance values from the plant surfaces and provides data on normalized difference vegetation index (NDVI) and normalized difference red edge index (NDRE). While a negative signal is typical of healthy plants, reduced NDVI and NDRE are associated with infected plants. Subsequent molecular analysis confirmed the data obtained by the optical sensor, thus demonstrating the great potential of this technique [137].

### 2.4. Nitrogenase Assays

#### 2.4.1. Nitrogen Fixation and Gas Chromatography

Nitrogen gas (N_2_), which makes up approximately 80% (by volume) of the air, cannot be used directly by plants to synthesize essential nitrogen-containing molecules, such as amino acids and nucleotides. Therefore, it must first be converted (fixed) into ammonia, through a process that requires a high input of energy to break the triple bond of N_2_ which is extremely stable [138]. Unlike the chemical synthesis of ammonia, which requires very high levels of temperature and pressure, biological nitrogen fixation operates at ambient temperature and pressure [139]. The energy for the biological fixation of nitrogen comes from the hydrolysis of large amounts of ATP (i.e., 16 moles of ATP are required for the fixation of each mole of N_2_; see reaction 1 below).

More than 100 million tons of fixed nitrogen are required annually to sustain global food production. Chemically produced fertilizers account for around half of this nitrogen supply, while most of the remainder is from diazotrophic (nitrogen-fixing) bacteria. Notwithstanding their effectiveness in increasing crop yields, chemical fertilizers have led to (i) pollution problems because of runoff from farmer’s fields and depletion of the nutrient reserves in the soil, (ii) increasing costs, and (iii) an enormous amount of the limited global energy supply needed to chemically synthesize nitrogen fertilizers. On the other hand, while eukaryotes cannot fix nitrogen a wide range of bacteria can fix nitrogen, and several of them have potential as crop fertilizers.

Bacterial strains able to fix nitrogen, which are most frequently used in agriculture, belong to a range of rhizobial genera and species [140]. These bacteria stained as Gram-negative, are flagellated, rod-shaped, and able to establish symbiotic relationships with legumes. Generally, each rhizobial species is specific to a limited number of plants and does not interact with plants other than its natural hosts. In addition to rhizobia, a wide range of free-living bacteria can fix nitrogen including the genera *Rhizobia*, *Ensifer*, *Bradyrhizobia*, *Mesorhizobia*, *Frankia*, some cyanobacteria, and some species of *Azospirillum*, *Azotobacter*, *Enterobacter*, *Chryseobacterium*, *Klebsiella*, *Bacillus*, *Paenibacillus*, *Sphingobacterium*, and *Pseudomonas*.

All known nitrogenases (the enzymes that fix nitrogen) are inhibited by oxygen and are composed of two protein components. Component I is a complex of two identical alpha-protein subunits (~50,000 daltons each), two identical beta-protein subunits (~60,000 daltons each), 24 molecules of iron, 2 molecules of molybdenum, and an iron–molybdenum cofactor, called FeMoCo [141]. Component II contains two alpha-protein subunits (~32,000 daltons each), which are not the same as the alpha-protein subunits of component I, and several associated iron molecules. The conversion of gaseous nitrogen to ammonia requires the combination of components I and II, the availability of magnesium and ATP, and a source of reducing equivalents with the overall reaction depicted below (reaction 1).

N_2_ + 8H^+^ + 8e^−^ + 16MgATP → 2NH_3_ + H_2_↑ + 16MgADP + 16Pi (1)

In addition to converting gaseous nitrogen into ammonia, the nitrogenase can also reduce the gas acetylene to the gas ethylene (reaction 2).

H—C≡C—H + 2H^+^ → H_2_=C=C=H_2_ (2)

**Figure 5:**
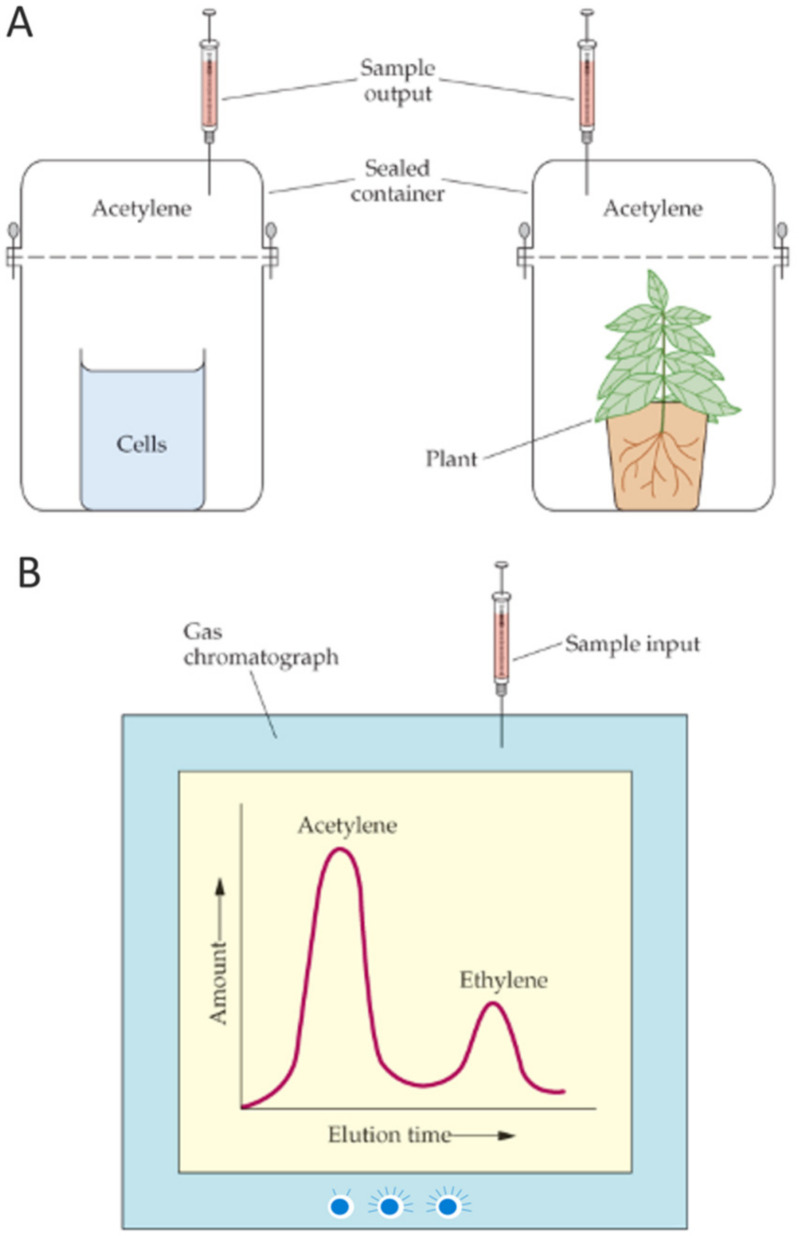
A gas chromatographic assay for nitrogenase activity based on the conversion of acetylene to ethylene by the nitrogenase enzyme. (**A**) The source of the nitrogenase enzyme, which may be bacteria growing in culture, bacteria associated with plant roots, or a purified enzyme preparation, is placed in a sealed container under an atmosphere of 90% argon or helium (both inert gases) plus 10% acetylene. (**B**) The test sample is incubated for various periods of time, with shaking, and 0.5–1.0 mL aliquots of the gaseous atmosphere are periodically withdrawn, using a fine-tipped syringe, from the sealed container. The contents of the gas-filled syringe are injected into a gas chromatograph and the levels of acetylene and ethylene are quantified. The amount of nitrogenase activity is proportional to the amount of ethylene that is produced in each time period.

The measurement, by gas chromatography, of ethylene production from added acetylene, as a function of time, provides a convenient assay for nitrogenase activity. This assay can be performed with intact cells in solution, bacteria associated with plant roots, crude cell extracts, or even highly purified enzyme preparations (Figure 5). Component I of the nitrogenase complex catalyzes the reduction of N_2_, while component II donates electrons to component I. In addition to these components, the activity of a functional nitrogenase depends on ~15–20 additional proteins (most of which are involved, either directly or indirectly, in shuffling electrons to component II or in the biosynthesis of FeMoCo which is a key part of component I. 

Nitrogenase activity is commonly assayed by monitoring the conversion of acetylene to ethylene and this assay has been used by many laboratories for more than 40 years. However, there are several drawbacks to this assay. These include: the cost and maintenance of a gas chromatograph, which is typically dedicated to this measurement; the difficulty of measuring low levels of nitrogenase activity, especially when the background level of ethylene in the atmosphere in many labs is relatively high and/or changing; and the difficulty of excluding all traces of oxygen from the assay. Thus, while many labs rely on this method, others have sought to develop and utilize alternative means of measuring nitrogen fixation.

#### 2.4.2. Viologen-Based Assay

**Figure 6:**
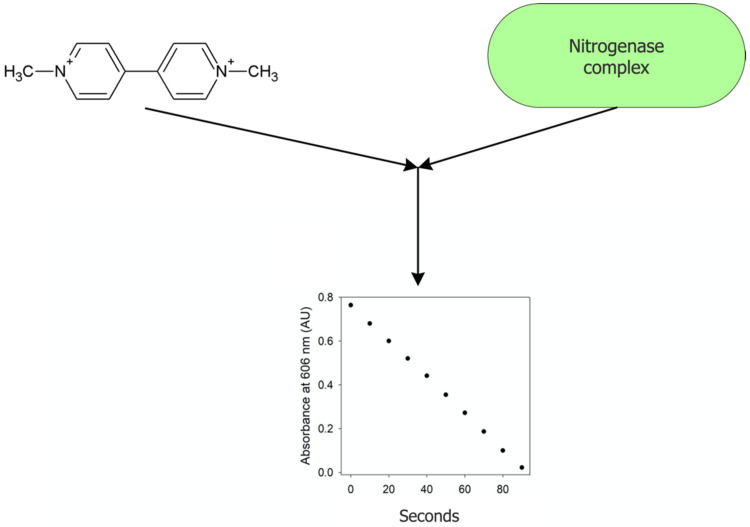
An assay for nitrogenase activity in which the absorbance changes to methyl viologen following its binding to a nitrogenase complex is monitored spectrophotometrically over time at a wavelength of 606 nm. The reaction is monitored in an argon environment, which is completely oxygen-free.

Some researchers have endeavored to avoid having to utilize a gas chromatograph and have used several alternative procedures to estimate nitrogenase activity. For example, at pH 7.0, in the presence of 6.7 mM MgCl_2_, 5 mM ATP, and an ATP regenerating system, nitrogenase interacts strongly and rapidly with the compound methyl viologen (Figure 6). Under these conditions, methyl viologen is rapidly oxidized, a reaction that can be followed spectrophotometrically at 606 nm. This reflects the catalytic reduction of protons to hydrogen gas, with the reaction occurring under an argon atmosphere to avoid any oxygen inhibition of the nitrogenase [142]. This procedure requires a sensitive spectrophotometer with the ability to rapidly add and mix the reaction components. It should be noted that very similar conditions may be employed to measure the activity of bacterial hydrogenases [143,144]. Thus, it is possible to utilize the interaction between methyl viologen and the nitrogenase enzyme and the subsequent color change as a direct means of monitoring the activity of the purified nitrogenase enzyme. With the conditions employed in this assay, instead of monitoring the color change, it might also be possible to monitor the evolution of hydrogen gas using a hydrogen electrode.

#### 2.4.3. ^15^N Dilution Method

When plants (white bean or soybean) were planted (in a test plot of 44 × 60 cm) some of the plots were amended with 0.8 g ^15^NO_3_, with the controls receiving an equivalent amount of N as ^15^N unenriched NH_4_NO_3_ [142]. At maturity, the plants were harvested, weighed, and dried. Following disruption and dissolution of the samples and subsequent conversion of all nitrogen into NH_3_ and then N_2_, the ^15^N:^14^N ratios of the gaseous N_2_ were determined by mass spectrometry. The proportion of the total amount of plant nitrogen that is derived from nitrogen fixation is estimated by comparing the ^15^N:^14^N ratios of the nitrogen-fixing plants to the ^15^N:^14^N ratio of the control plant (i.e., no added nitrogen=fixing bacteria added). This older method is both accurate and effective, however, it requires access to an expensive mass spectrometer. Moreover, the preparatory steps are complex and time-consuming. Interestingly, in one early study [145], the ^15^N dilution method appeared to be considerably more sensitive (for both white bean and soybean) than the acetylene reduction assay. In speculating why this might be the case, Smith and Hume [145] pointed out that, among other limitations of the acetylene assay, “acetylene can be inhibitory to nitrogenase activity, resulting in an underestimation of N_2_ fixation rates by the acetylene reduction assay”.

#### 2.4.4. Modified Acetylene Reduction Assay

It was recently pointed out [146] that the acetylene reduction does not necessarily reflect the nitrogenase activity of a particular bacterium either within a root nodule or bound to a plant root. That is, nitrogenase assays using the standard acetylene reduction assay are typically carried out on free-living nitrogen-fixing bacteria that have been isolated, purified, and then grown in a laboratory in the absence of any host plant. It is argued that such an assay is not a true reflection of the nitrogenase activity that exists when the bacteria are directly associated with the roots of a plant. The direct interaction between bacteria and plant roots enables bacteria to receive various carbon- and nitrogen-containing molecules, some of which modulate nitrogen fixation, from the root exudates of the plant. Thus, it is important to assess the behavior and activity of the specific bacterial strains that are intended for agricultural use in the presence of specific plants since, when they are developed for agriculture, these bacteria will be used in conjunction with these plants.

To identify bacterial strains that are prolific nitrogen fixers when they were associated with plants, Haskett et al. [146] grew germinated barley seeds (previously sterilized to remove any surface adhering microbes) in sterilized 100 mL glass bottles (one germinated seed per bottle) filled with 50 mL of industrial-grade sand and 15 mL of N-free and C-free rooting solution. At various time intervals, nitrogenase activity was assessed by the acetylene reduction assay. With this system, nitrogenase activity was observed only in the presence of both a plant and added bacteria, with the highest level of nitrogenase activity observed when the oxygen level was decreased to 1%. When eight different nitrogen-fixing bacteria were compared, the maximum nitrogenase activity was observed with strains of *Azoarcus olearius* (isolated from oil-contaminated soil in Taiwan) and *Pseudomonas stutzeri* (isolated from a rice rhizosphere in Southern China). Interestingly, when the bacterial strains were tested this way, they showed a very wide range of nitrogenase activity. Therefore, as intended, this approach should help agronomists to select diazotrophic bacterial strains that are likely to function most efficaciously with any designated plant species.

### 2.5. Specialized Growth Chambers

#### 2.5.1. Rhizotrons and Mini Rhizotrons

According to the definition given by Kloepper and Kaspar [147], the word rhizotron indicates “a facility or building for viewing and measuring underground parts of plants through a transparent surface”. More in detail, rhizotrons are subterranean rooms, laboratories, or plane containers with transparent glass or plastic walls allowing the visualization of roots growing in soil. In addition, rhizotrons are equipped with sensors measuring in real time, the temperature, oxygen concentration, soil water activity, pH and many other parameters.

Minirhizotrons include transparent tubes which are placed in soil and equipped with fiber-optic borescopes or video cameras [148]. This kind of tool allows the observation and measurement of the root growth dynamic during short time intervals, also in open field-like conditions, maintaining the integrity of the living root system.

The first rhizotron was built in 1961 in Kent, UK with the aim of analyzing the changes in the root development of fruit trees according to seasonal changes [149]; since that time many other rhizotrons have been constructed all over the world. The two main drawbacks of this instrument are the installation and maintenance costs and the lack of three-dimensional images when visualizing root system architecture. In fact, the quality of images obtained from rhizotrons and minirhizotrons is a key factor in the efficient evaluation of root development. There are several cost-effective techniques for root imaging in the field such as tracing onto a transparent plastic sheet, scanning with a flatbed or handheld scanner), or with a Smartphone scanner application [150]. Although the availability of these tools represents an advantage of rhizotrons over minirhizotrons, the production of 3D images can be obtained by more expensive and more accurate recently developed techniques such as magnetic resonance imaging (MRI) [151], X-ray computed tomography (CT) [152], or neutron tomography [153].

On the other hand, minirhizotrons are much less expensive than rhizotrons and, at the same time, provide an accurate visualization of the root architecture dynamic in soil and soil-less systems [154]. This method is completed by the production of in situ images that need to be processed by manual or semi-automatic segmentation. During manual segmentation the operator analyzes the images, identifying and classifying each root by visually inspecting all images; this often results in the occurrence of many errors [155,156]. In this case, semi-automatic segmentation is based on the combination of an automated segmentation algorithm under the control of human–computer interactions.

In a recent study, a comparison among three cultural methods (hydroponic system, plane, and cylindric rhizotrons), two bi-dimensional (hydroponic system and rhizotrons), and one three-dimensional imaging techniques, consisting of neutron tomography were performed on grapevine cuttings. The results obtained highlighted that, conversely to rhizotrons, the hydroponic system does not allow the measurement of root traits during a specific time span. The 3D neutron tomography system was the most efficient method to evaluate the volume of the root system. Moreover, the authors developed an image analysis script for plants growing in rhizotrons allowing for the performance of an automatic root architecture analysis especially focused on adventitious roots in 3–5 min [157].

#### 2.5.2. Rhizobox

The root system is the hidden half of the plant, serving a multitude of functions such as the uptake of water and nutrients, the establishment of symbiosis with various microorganisms, and the anchorage of the plant in the soil [120]. For these reasons, the way in which a root develops generally reflects the health status of the plant. Therefore, growing plants under controlled conditions on a small scale is a key step in the identification and characterization of PGPB from bacterial strains previously selected from environmental matrices.

**Figure 7:**
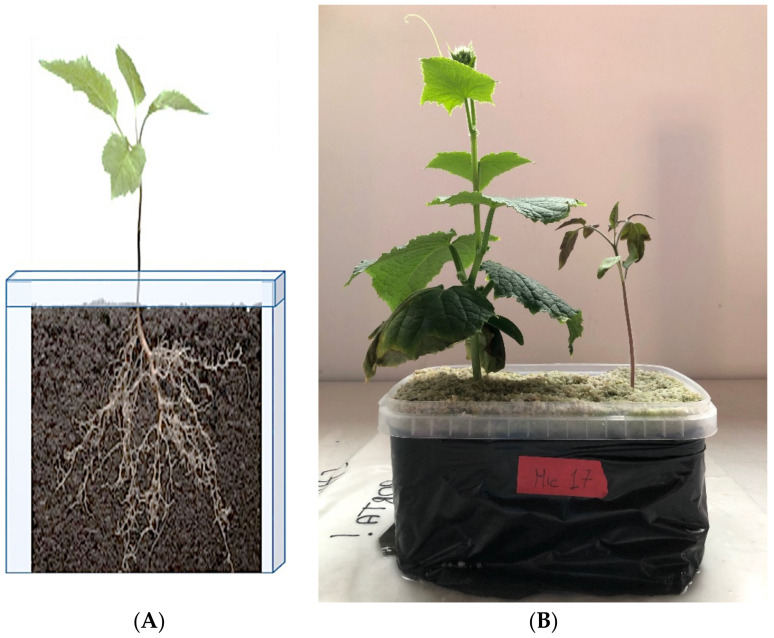
(**A**) Plant growth in a rhizobox with a transparent side allowing one to observe the root growth dynamic; (**B**) co-cultivation of cucumber and tomato plants in a rhizobox; the transparent container is wrapped with black foil to prevent the light from reaching the root. In this case, the rhizobox contained quartz sand added with arbuscular mycorrhizal fungi in order to monitor the formation of the mycorrhizal network in the two plant species.

Rhizoboxes are containers of different sizes and shapes, allowing researchers to continuously monitor root growth during an experimental time course without causing any disturbance to the plant. Petri dishes, half-cylinders, flat rectangular plots, and also plastic CD cases [158,159] filled with semi-solid nutrient media, soil, sand, or other substrates, can be used to build a rhizobox (Figure 7A). Usually, rhizoboxes are incubated in an inclined position to allow the root to grow as close as possible to the transparent side of the rhizobox. Obviously, the size, shape, material, and color of the container can affect plant root development [158]. The use of a rhizobox may be considered as an experimental setup whose complexity is intermediate between root phenotyping on artificial media such as filter paper and root imaging in open field conditions performed with minirhizotrons [160].

The use of rhizoboxes is quite common and several experiments have been conducted with this tool to characterize root growth dynamics, especially in plants exposed to abiotic stresses [161,162], in plants interacting with various microorganisms [163,164], or in plants subjected to different nutritional conditions [165,166].

An analysis of the images of roots growing in a rhizobox is probably the major bottleneck of the whole procedure of root phenotyping. In fact, in rhizoboxes only a limited portion of the root system is visible, especially if it is filled with non-transparent media [167]. Semi-solid transparent media represents a means of allowing the monitoring of root growth through optical sensors or laser scanning. However, it should be considered that root exposure to light can significantly affect root development and may trigger the expression of genes regulated by light thereby enhancing ROS production. Thus, rhizoboxes containing transparent media should be covered to prevent light from reaching the roots (Figure 7B), thus limiting the possibility of continuously following root development over time [168].

While several high-sensitivity image analysis methods have been developed such as MRI (Magnetic Resonance Imaging) or X-ray tomography, optical imaging tools based on digital cameras or a scanner, are used more frequently because of their lower cost and greater ease of use [169].

#### 2.5.3. Split-Root Systems (SRS)

**Figure 8:**
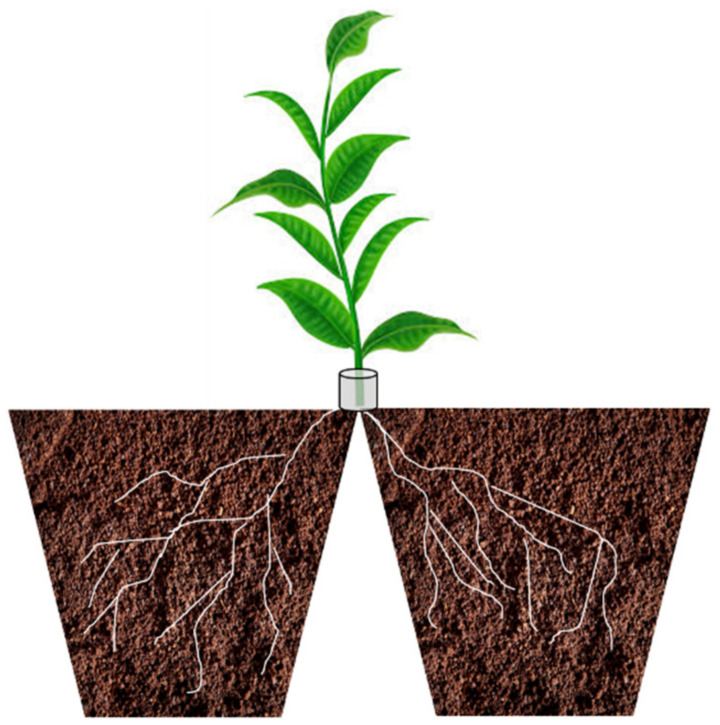
Image of a split-root system where the root has been longitudinally cut and grown into two compartments isolated from one another. This system allows one to assess root development in two separate compartments where different environmental (i.e., presence or not of salt stress) or biological (i.e., presence or not of phytopathogens) stimuli are applied.

The use of split root systems allows the cultivation of a plant with its root growing into separate and isolated compartments. Usually, the method follows seed germination and seedling development with the emergence of the primary root. Then, there are two strategies that can be followed. The primary root is longitudinally cut and each section is allowed to grow into separate pots/containers or the tip of the primary root is removed triggering root branching so that lateral roots are located in different pots/containers [170,171,172] (Figure 8). The plant species and the objectives of the experiment will dictate which approach is taken. The roots can be grown in two separate pots, in a single pot or in Petri dishes containing a plastic divider, in a piping elbow, or in twin-tube systems [173]. The main advantage offered by SRS is the possibility of employing two different treatments on the root systems while sharing the same epigeous portion of the plant.

The first application of this simple but efficient tool dates back to 1943 when Long [174] used SRS to study the effect of salinity on the water and nutrient uptake in tomato plants. Following this pioneering work, several studies employed SRS to analyze different aspects of plant metabolism, however, the main use of this system has been to discriminate between local and systemic effects on plants and to study their regulation. For example, a specific treatment (i.e., salinity stress, contamination of the substrate with heavy metals, or inoculation of the roots with rhizobia or AMF) is given to the roots growing in one pot and the response to the treatment is monitored on the side of the root not exposed to the stimulus. If the treatment induces a response that is systemically regulated, the untreated part of the root will show an evident response. However, if the treatment stimulates a local response, the specific response will occur only in the root exposed to the stimulus [172].

Even though plants can easily adapt to this artificial system, it should be considered that plants grown in SRS can show diverse tolerance to the exposure to stress, similarly to what was previously observed in grafted plants [175].

## 3. Manipulation of the Native Rhizosphere Microbial Community

### 3.1. Releasing PGPB into the Environment

Regardless of how effective a PGPB strain is under controlled laboratory conditions, its ultimate utility depends upon how well it performs in the field. In addition to a strain being effective at promoting plant growth in the field, in different countries and jurisdictions within countries, a range of government regulations control the deliberate release of these bacteria into the environment. In many jurisdictions, only the release of genetically engineered bacteria is regulated, while in many others even the release of native unmodified bacterial strains is subject to governmental oversight.

Regardless of whether the microorganisms deliberately introduced into the environment are wild-type or genetically modified bacterial strains, it is required that they are not harmful to the environment, plants, animals, and humans. Consequently, several factors must be considered prior to deliberately releasing PGPB into the environment. These factors include the survival rate once introduced in the environment; the capability to grow and proliferate in different environmental conditions; the probability that DNA from the released PGPB will be transferred to other microorganisms in the surrounding environment through horizontal gene transfer; the capability of the released PGPB to spread in the environment; the possible risk represented by the released PGPB for any other organisms in the environment [176,177,178].

Bacterial survival varies considerably in different soil types with survival being much greater in nutrient-rich soils and in soils with high clay content. PGPB associated with plant roots can persist for months, while in the bulk soil the density of the same bacterial strain may decrease by many orders of magnitude [179,180]. Endophytic PGPB, within a plant’s tissues, can generally persist for long periods of time, often related to the life of the plant host. Several soil parameters such as pH, temperature, compaction, and oxygen content can affect bacterial survival in the soil. Genetically modified PGPB usually show a lower survival and replication rate in the environment compared to the native non-transformed forms of these bacteria. This is likely a consequence of the metabolic load [181] that is imposed on genetically transformed cells by the expression of foreign DNA.

Bacterial genes, whether the genes are native to the host bacterium or introduced by genetic engineering, can readily be transferred to other organisms in the environment, a process that has been going on for many millions of years [182,183,184]. With the complete DNA sequencing of hundreds of thousands of bacterial genomes, it has become clear that most soil bacteria contain several stretches of DNA that appear to have been transferred from other soil bacteria mainly through horizontal gene transfer. If genetically manipulated PGPB are deliberately released into the environment, it is essential that (i) the behavior of these bacteria is monitored and (ii) the PGPB strains are engineered so that it is less likely that they will transfer their DNA to other bacteria in the soil. This may be done by ensuring that any genetic alterations to a PGPB strain are limited to the bacterial chromosomal DNA and not to any, much more easily transmissible, plasmids.

According to European Union (EU) rules, “any organisms altered by recombinant DNA techniques and techniques involving the direct introduction into an organism of heritable material prepared outside the organism including micro-injection, macro-injection, and micro-encapsulation” are subject to a very high level of government as well as community scrutiny before they can be used in the environment [185]. This rule limits the deliberate release of genetically engineered PGPB in the environment within the EU and the importation of any transgenic PGPB into the EU. However, the EU definition of genetic modification does not include traditional or CRISPR mutagenesis [186]. Here, it is necessary to keep in mind that these rules may be continually updated and changed and that the guidelines/rules established by the EU are a mirror of the approach taken in many other countries around the world. If scientists can genetically engineer superior PGPB strains, then it is likely that foreign genes or gene fragments will be incorporated into PGPB chromosomal DNA using either homologous recombination or CRISPR techniques.

During the past 30 years, several transgenic rhizobia strains have been introduced into the environment [187] in both the US and the EU (Table 2). Most of these releases had the objective of monitoring either the environmental functioning of potentially improved PGPB strains or the environmental fate of those strains.

### 3.2. CRISPR Fundamentals

CRISPR (Clustered Regularly Interspaced Short Palindromic Repeats) methods of modifying chromosomal DNA are based on a natural prokaryotic system that protects bacteria against invasion by foreign DNA from either bacteriophages or foreign plasmids. The CRISPR-Cas system (where Cas is a nuclease enzyme that uses CRISPR sequences to recognize where to cleave DNA strands that are complementary to the CRISPR sequence) can be adapted to introduce or replace genes in the genomes of a wide variety of organisms, both prokaryotes, and eukaryotes, and also to edit genomes, that is, remove or alter targeted nucleotides [194,195,196,197].

Because of its relative simplicity compared to systems in other bacteria, the CRISPR-Cas system from *Streptococcocus pyogenes* has been developed for use as a genome engineering tool. In the natural *S. pyogenes* system, two RNA molecules, crRNA and transactivating crRNA (tracrRNA) form a crRNA:tracrRNA hybrid that directs the Cas9 endonuclease to the target site. For ease of use in genome engineering, the two RNAs are combined into a single guide RNA (sgRNA). Following binding to the target DNA sequence, the endonuclease makes a double-stranded break in the target DNA. This cleavage activates the cellular systems for DNA repair either by homologous recombination, in which DNA sequences with sufficient similarity are exchanged or by non-homologous end joining, in which sequences are deleted or inserted. The cellular repair systems can be harnessed to disrupt, insert, or replace a DNA sequence at a targeted site.

**Figure 9:**
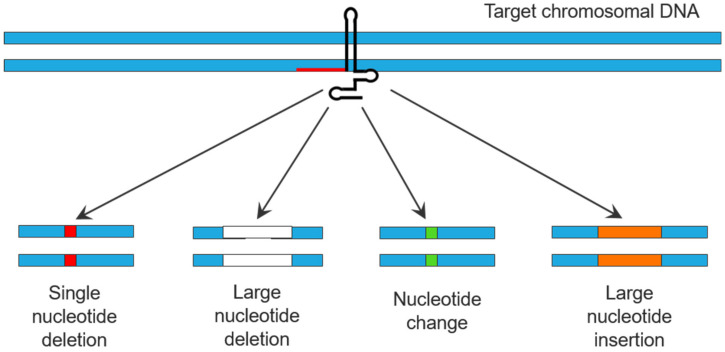
Schematic representation of a CRISPR molecule bound to bacterial chromosomal DNA. For simplicity of presentation, the Cas9 protein molecule is not shown. The CRISPR molecule is positioned opposite the target sequence on the chromosomal DNA by the (80–100 nucleotide long) sg DNA shown in red. A single or a multiple (i.e., large) nucleotide deletion in the middle of a gene yields a knockout mutation that no longer encodes the product of that gene. An altered or changed nucleotide generally yields a missense mutation that encodes an altered version of the original protein. A large nucleotide insertion may be used to introduce an entire gene.

In its simplest form, the CRISPR genome modification technology consists of a single RNA guide molecule (sg) ~80–100 nucleotides long that matches a portion of the target DNA sequence, together with the remainder of the CRISPR molecule, and the endonuclease Cas9 from the bacterium *S. pyogenes* (although Cas endonucleases from other bacteria have also been utilized). The sgRNA and the Cas9 endonuclease form a complex in which the sgRNA guides the complex to its target DNA and the nuclease activity of Cas9 then cleaves the target chromosomal DNA in both strands (Figure 9). With the CRISPR/Cas9 system, only the nucleotides in the sgRNA need to be changed to confer different target specificities (i.e., where the target DNA is cleaved), and therefore it is possible to alter several genes at the same time, potentially knocking out redundant or parallel pathways within a bacterium.

### 3.3. CRISPR Modified PGPB and Phytopathogens

The CRISPR/Cas system has been used several hundred times to modify plant genomes, however, it has been used relatively infrequently in the modification of PGPB chromosomal DNA. Nevertheless, the CRISPR/Cas system modification of PGPB may be used (i) to tag bacteria so that their behavior in the soil and interaction with plant roots and with other soil microbes may be monitored over time; (ii) to generate PGPB mutants that are devoid of a specific targeted activity so that the activity involved with the interaction with plants may be assessed in a laboratory setting; (iii) to generate PGPB mutants that overexpress a protein encoded by an endogenous PGPB gene; (iv) to synthesize a variant of an endogenous PGPB gene; or (v) to introduce an exogenous gene into a PGPB genome.

In one study, Yi et al. [198] demonstrated that *Bacillus subtilis* strain HS3 which synthesizes the antifungal lipopeptides fengycin and surfactin and emits the volatile organic compound 2,3-butanediol, selectively colonizes the root hairs of the grass *Lolium perenne* in a hydroponic system. To assess whether the above-mentioned lipopeptides were responsible for the antifungal activity of this bacterium, these researchers used the CRISPR/Cas system to knockout the bacterial gene *sfp* which encodes 4′-phosphopantetheinyl transferase, an enzyme necessary for the synthesis of the aforementioned lipopeptides. When the *sfp* gene was knocked out, the antifungal activity of this strain against the fungal pathogens *Rhizoctonia solani* and *Fusarium colmorum* was abolished confirming the importance of fengycin and surfactin in the antifungal activity of this biocontrol strain. In addition, these researchers used the CRISPR/Cas system to knockout the synthesis of 2,3- butanediol. However, in this case, the mutated PGPB strain was unaffected in its ability to promote plant growth suggesting that mechanisms other than the synthesis of 2,3-butanediol likely were responsible for the ability of this bacterium to promote plant growth.

Like the approach that was taken by Yi et al. [198], several research groups reported modifying various phytopathogen strains with the CRISPR/Cas system in order to develop mutations in specific phytopathogen genes. The strains with those mutations were then tested for their ability to function, thereby proving their involvement in specific pathogen activities. Thus, the CRISPR/Cas system has been utilized in the mutagenesis of *Fusarium oxysporum*, *Fusarium proliferaturm*, *Alternaria alternata*, *Phytophthora* spp., *Sclerotinia sclerotiorum*, and *Colletotricheum sansevieriae* [199,200,201,202,203]. These experiments were performed as a means of better understanding the pathogen infection process.

Furthermore, it was shown that when the CRISPR/Cas system was used to delete the *ace1* gene (a repressor of cellulase and xylanase synthesis) from the plant beneficial fungus *Trichoderma atroviride*, the expression of four polyketide biosynthetic genes was induced and the biocontrol activity of this fungal strain against the phytopathogens *Fusarium oxysporum* and *Rhizoctonia solani* was enhanced [204]. The high level of increased biocontrol by this *Trichoderma* strain was attributed to significantly increased levels of the cell wall-degrading cellulase and xylanase enzymes as well as the increased level of the core polyketide antibiotic. This environmentally benign use of the genome-edited *Trichoderma* strain may lead to an eco-friendlier mode of plant disease management.

The acidic heteropolysaccharide pectin is a major constituent of the cell wall of nearly all higher plants. The main component of pectin is galacturonic acid and some of the galacturonic residues in pectin are O-acetylated. The enzyme pectin acetylesterase, which is involved in the enzymatic deacetylation of pectin, is commonly found in many phytopathogenic oomycetes (fungus-like eukaryotes). In particular, the oomycete *Peronophythora litchii* is the most destructive pathogen of lychee (*Litchi chinensis*), a tropical tree that produces small fleshy edible fruits, popular in Southeast Asia since the 11th century. Of the 38 pectin acetylesterases produced by this pathogen, two pectin acetylesterase genes were separately knocked out using the CRISPR/Cas system [205]. One of these engineered mutant strains was significantly less able to invade lychee plants compared to the wild-type *P. litchii* strain. Whether this genome-modified oomycete can act as a biocontrol strain (possibly outcompeting the pathogenic wild-type strain) remains to be determined.

When scientists develop PGPB strains (in the lab) that will eventually be used in the field, it is generally necessary to label those bacteria before they are released into the environment so that their behavior in the field can be monitored and assessed. Some of the labels and techniques that have been employed to monitor various bacterial strains include the *lacZ* gene, the *gusA* gene, the *luxAB* genes, fluorescent in situ hybridization, and quantitative PCR. While these techniques work quite well under laboratory conditions, they are often much less effective under field conditions. To get around any constraints or limitations that may exist when using the above-mentioned laboratory approaches, one group of scientists first identified CRISPR *loci* that existed in a strain of *Azospirillum* that they were studying [206]. CRISPR *loci* are short palindromic repeats interspersed with spacer sequences found within the chromosomal DNA of an organism. Identifying these sequences is relatively straightforward given the fact that the complete genomic DNA sequence of thousands of PGPB is currently known, with more sequences regularly being added to this database. Moreover, within a given CRISPR *locus*, the repeats are nearly identical in both length and sequence. Once having identified the unique CRISPR *locus* sequences carried by a particular bacterial strain, it is possible to subsequently identify each unique strain by PCR amplification of one or more CRISPR *loci*. With this procedure, it is not necessary to label or introduce any foreign DNA into the PGPB strain. Thus, the behavior of native unmodified PGPB strains that are utilized in the field can readily be monitored.

The bacterium *Clavibacter michiganensis* is an aerobic non-sporulating Gram-positive phytopathogen that is responsible for significant crop losses worldwide. One subspecies of this bacterium (i.e., *michiganensis*) is the causal agent of bacterial canker of tomato, a disease that can cause dramatic losses to tomato production; another subspecies (*sepedonius*) only affects potatoes, causing the rot of vascular tissue inside potato tubers, and a third subspecies (*insidiosus*) causes damage to alfalfa plants. Notwithstanding the rather limited number of studies of this phytopathogen, it has recently been shown that cell-wall-degrading enzymes and serine proteases are key components in the development of plant wilt and canker [207]. Following the discovery of CRISPR *loci* in the genome of *C. michiganensis*, researchers developed a gene-editing strategy for this bacterium based on the CRISPR/Cas system [208]. The introduction of the *codA::upp* cassette into a transformation vector (directed towards a CRISPR *locus*) makes the transformants sensitive to the lethal effects of the compound 5-fluorocytosine. The *codA::upp* cassette encodes a fusion protein of *E. coli* cytosine deaminase (the *codA* gene) and *E. coli* uracil phosphoribosyltransferase (the *upp* gene). Bacteria that express cytosine deaminase activity convert the compound 5-fluorocytosine into 5-fluorouracil which is, in turn, converted to 5-fluoro-dUMP by the enzyme uracil phosphoribosyltransferase. The 5-fluoro-dUMP irreversibly inhibits cellular thymidylate synthase, and nucleic acid synthesis, ultimately causing cell death. The introduction of the *codA::upp* cassette into the CRISPR *locus* of this pathogenic bacterium facilitates the counterselection of transformants without the use of a selective antibiotic when the transformation is followed by plating onto minimal medium containing 5-fluorocytosine. This system should facilitate the development of a more detailed understanding of this, otherwise difficult to study, pathogenic bacterium.

The problems caused by the need to safely and effectively dispose of various types of environmental wastes are enormous. For example, it has been estimated by the U.S. E.P.A. that more than 40 million tons of hazardous waste are produced each year in the United States. In the past 20–30 years, scientists have begun testing the ability of different plants to facilitate the remediation of polluted soils, that is, the process of phytoremediation. This developing technology is perceived as a clean, effective, and relatively inexpensive means of addressing the problem of environmental cleanup. However, phytoremediation also has some technical drawbacks, as very few plant species can tolerate high concentrations of most environmental contaminants including both metals and organics. However, certain soil bacteria, including many PGPB, can assist plants in the process of phytoremediation [209,210]. This may occur by a variety of mechanisms including facilitating the breakdown of some organic compounds and lowering the stress level of plants involved in phytoremediation. In addition, it is possible to genetically engineer both plants and bacteria to function more efficiently in the process of phytoremediation. This genetic manipulation may include either the introduction of foreign genes or the alteration, by CRISPR/Cas, of already existing plant or bacterial genes [211]. However, while the CRISPR/Cas system has been utilized to make a small number of plants more effective in phytoremediation [212], until now, there are no reports of using the CRISPR/Cas system to modify bacteria that facilitate phytoremediation.

### 3.4. Inoculant Encapsulation

There are a huge number of scientific papers in the literature regarding the selection of new PGPB. Notwithstanding the effectiveness of these bacteria under laboratory conditions, their utilization in the field remains only a very small fraction of current agricultural practice at a global level, well below their potentiality [213]. This is mainly due to the inconsistent and variable performance of PGPB when they are applied in open field conditions, thus hampering their commercialization on a large scale [214,215,216]. At the base of this issue are: (i) the survival of PGPB once introduced into the bulk soil or rhizosphere, (ii) the compatibility of the PGPB with the inoculated plant and soil parameters, (iii) the interrelationships occurring between the introduced PGPB and the resident soil microflora [217], and (iv) the pivotal role played by the method of inoculant formulation, which is related to the compatibility and stability of the carrier chosen [218,219,220].

Bacterial encapsulation, by creating an envelope ensuring cell protection, allows the controlled release and maintenance of cell functional traits, thus providing a useful tool to overcome environmental difficulties and enlarge the potential range of applications [221]. There are several advantages of bacterial encapsulation: (i) the bacterial cells are enclosed in a protected environment extending bacterial survival during storage; (ii) in a context where competition with autochthonous bacteria is high, bacterial root colonization is favored; and (iii) the possible predation by soil organisms of the bacterial inoculant is drastically reduced.

Different chemical (molecular inclusion, interfacial polymerization), physical (spray drying, extrusion), and physicochemical (coacervation, liposomes, ionic gelation, inverse gelation, beads by oil-entrapped emulsion) encapsulation technologies are available. Encapsulation is typically combined with one of several carriers such as synthetic polymers, biopolymers, and organic/inorganic materials [222,223]. The compatibility of the carrier with bacterial cell survival is the main issue to be considered in this process so very often, the carrier is a food-grade material. For example, a wheat-gluten matrix known as Pesta has been used to formulate, in granular form, strain *Pseudomonas trivialis* X33d, a bioherbicide able to control the growth of the weed great brome (*Bromus diandrus*) [224]. More recent attention has been primarily focused on the development of encapsulation techniques based on carriers allowing a slow and gradual release of bacterial cells into the soil [221,225].

**Figure 10:**
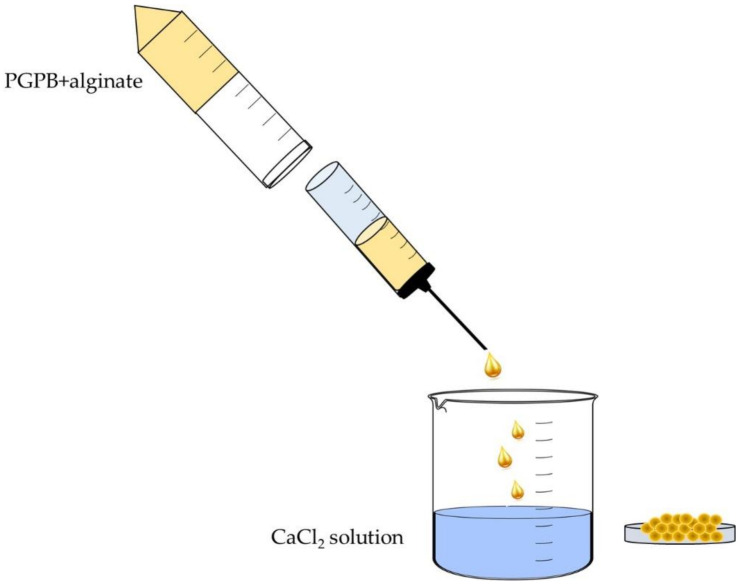
Schematic image of the procedure of bacterial encapsulation in alginate. The first step consists of preparing a solution of the PGPB cells in sodium alginate. Then the suspension is dropped by a needle into a calcium chloride solution to allow the alginate gelification mediated by the substitution of Na with Ca. As a result, bacterial cells remain entrapped in the calcium alginate capsule, ensuring a protected environment and long viability of the strain.

Among the available encapsulation techniques for PGPB, due to its low cost and simple execution, extrusion is one of the most popular [226,227]. This method is defined as the change of an emulsion of an active material and wall material under high pressure. During the procedure, the bacterial cells are suspended in a matrix polymer at a high temperature. While the extrusion device releases the bacterial cells, the exterior pore releases the envelope to liberate bacterial cells covered by a wall-like compound [222,228]. Bacterial encapsulation through extrusion is usually performed inside hydrogel particles derived from biopolymers or food-grade biopolymers, especially natural polysaccharides or proteins including starch, dextrin, gum Arabic, malt, pectin, chitosan, alginates, and legume proteins [229,230]. These biopolymers have a high degree of biocompatibility, biodegradability, reliable amphiphilic ability, water solubility, and emulsifying and forming properties [231]. The cell encapsulation procedure can be described in three steps. A bacterial suspension is mixed in an aqueous biopolymer having gelling properties and then extruded into a gelling environment via a small nozzle to allow the formation of droplets of the biopolymer carrying the bacterial cells inside. Finally, these droplets are stabilized against dissociation or aggregation (if alginate is used as biopolymer, the stabilizer is represented by a CaCl_2_ solution) (Figure 10). The hydrogel drops are then collected, rinsed, and dried [232].

However, the integrity of bacterial cells encapsulated in sodium alginate is often not fully preserved due to the low strength of the net. In fact, the mechanical strength of the alginate beads’ net can be improved by adding polymeric molecules such as polyvinyl alcohol (PVA). This strategy has been recently followed to prepare a PGPB inoculum of *Bacillus pumilus* G5 used to treat Japanese morning glory (*Pharbitis nil*) cultivated under drought and salt stress. Microbeads containing strain G5 increased the density of the rhizosphere bacterial culturable fractions, as well as the amount of several enzymatic activities, and improved plant growth parameters under stressful conditions [233].

#### Brief Overview of Some Encapsulation Techniques

Molecular inclusion results from the interaction between substances where the smaller molecule adapts to the net created by the other one. A typical example of this is the use of cyclodextrins, having a hydrophobic cavity whose size is appropriate to host a variety of molecules. However, this method is not suitable for bacterial cell immobilization due to their relatively large size [222].

Interfacial polymerization is based on the creation of an emulsion where the bacterial cells are the aqueous, discontinuous phase and an organic solvent represents the continuous phase. Following the addition of a biocompatible reactive material that is soluble in the organic solvent, the formation of droplets containing bacterial cells occurs. To avoid cell toxicity of the organic phase, an alginate suspension is dripped into a chitosan solution, and the resulting mixture is further dripped into a solution of chitosan (acetic acid 1% at pH 4) under continuous stirring. The use of this method to encapsulate bacterial cells is limited due to factors such as the possibility of high pH and toxic chemicals [234].

Spray drying is one of the most common encapsulation processes in large-scale microbial formulation ensuring a low cost and, at the same time, high quality of the final product. The bacterial cells are mixed with a carrier able to create an emulsion. The suspension is then homogenized, atomized, and sprayed into a chamber with a high temperature thereby causing the solvent to evaporate and leading to the development of microcapsules. However, this method is not well adapted to bacterial cell encapsulation due to the dehydration steps included in the process, and the high temperature needed to allow solvent evaporation which may be deleterious to bacterial cells [221].

Coacervation is based on the formation of a liquid rich in the polymer phase that is in equilibrium with another liquid phase defined as coacervate [235]. This process can be simple when a dissolved polymer interacts with a low molecular weight compound (i.e., gelatin with alcohol or sodium sulfate) and forms a complex if the interaction involves two polymers having opposite charges.

Liposomes are defined as microcapsule-like structures where the external membrane is represented by one or more hydrated bilayers which surround the active material in the internal space [236]. Liposomes are typically used to encapsulate DNA or RNA, or various drugs, rather than bacterial cells. However, the technology based on liposomes is quite expensive and is therefore generally used for processes occurring at a laboratory scale.

In ionic gelation, a drop of an aqueous suspension containing the bacterial cells is mixed with sodium alginate which is dropped into a solution of calcium chloride leading to the formation of an envelope of calcium alginate [237]. When the capsule is added to a sodium citrate solution, the calcium is solubilized, and the internal portion of the drop faces an ungelling event regulating the thickness and size of the wall-like structure. Due to the size of the needle used to drop the bacteria plus alginate suspension, the final bead dimension is usually measured in millimeters. However, micron and nano-sized microcapsules have also been produced using a solution of 1% chitosan in acetic acid at pH 4 [238,239].

Reversing the order of the ionic gelation procedure, inverse gelation includes dropping a calcium suspension into an alginate solution, thereby creating aqueous-core calcium alginate capsules [240,241].

Regarding the material used for PGPB encapsulation among biopolymers alginate, xanthan gum, starch, pea proteins, humic acids, bentonite, and skim milk have been widely used [242,243,244,245]. Synthetic polymers are less suitable for bacterial encapsulation due to their low biocompatibility related to the use of organic solvents during the procedure [225].

More recently, the development of nanomaterials (nanotubes, nanowires, fullerene derivatives, and quantum dots) has opened new bacterial encapsulation possibilities for use in agriculture [246]. Obviously, due to the size of the bacterial cells, nanocapsules are too small for cell encapsulation. However, it has been shown that SiO_2_ nanoparticles added to alginate/gelatin encapsulated PGPB boosted plant resistance against the phytopathogenic fungus *Sclerotium rolfsii* by hampering the pathogen’s colonization inside the plant tissue [247]. Similarly, nanoparticles containing metabolites synthesized by *Pseudomonas fluorescens* VUPF5 and *Bacillus subtilis* VRU1 increased the root length and proliferation of pistachio rootstock, thus facilitating its commercial utility [248].

## 4. Summary and Conclusions

The goal of all studies of how different plants respond to plant growth-promoting bacteria is the development of safe and efficacious bacterial inocula that can be used to facilitate the growth of plants under a wide variety of environmental conditions and on a large scale. A detailed understanding of the mechanisms employed by plant growth-promoting bacteria is absolutely essential for the successful commercialization of these bacteria and for the eventual replacement of potentially harmful chemicals (both to the environment and to people) in producing the food that we eat. To better understand the many mechanisms used by plant growth-promoting bacteria and to document the detailed effect of these bacteria on treated plants, scientists have developed and utilized a very wide range of techniques. These techniques employ both simple and highly sophisticated approaches including developing an understanding of the bacterial and fungal microbiomes that exist in the soil; sequencing the complete genomes of plant growth-promoting bacteria as a means of understanding how key genes are regulated and how various genes interact with one another; characterizing the mRNAs and proteins synthesized by different plant growth-promoting bacteria under a range of environmental conditions, editing or modifying the existing genes within a plant growth-promoting bacteria with the idea of improving the behavior of that bacterium as an adjunct to plant growth; developing approaches wherein plant growth-promoting bacteria are encapsulated as a means of stably and efficiently delivering them to their target plants; assaying key biological functions of various plant growth-promoting bacteria, in particular nitrogen fixation; using sophisticated image analysis methodology to assess the functioning of plants that have been treated with plant growth-promoting bacteria; and the development of specialized (both large and small) chambers to monitor the growth of plants treated with plant growth-promoting bacteria.

The more that we understand the fundamental functioning of plant growth-promoting bacteria, the more likely we are to be able to effectively use these bacteria in our future agriculture.

## Figures and Tables

**Table 1:** The table reported the advantages and limits of each approach taken alone. In order to overcome the limits, an integrated approach is important.

	Advantages	Limitations
Next-generation sequencing (NGS)	Possibility to identify in the same analysis all the species present in the sample and so also the unculturable strains.	No information regarding the activity of the identified bacteria and also the role of each species/interaction with other species only predictive data
Whole metagenome shotgun sequencing	Information regarding all the genomes present in the microbial communities	No information regarding the expression of the different genes
Metatranscriptomic	Detailed information regarding the transcripted genes (RNAs).	Low content of RNA in soil. RNA is a molecule with low stability. No information regarding the enzyme translation.
Metaproteomic	Detailed information regarding proteins and so the effective microbial work in the soil. Information regarding the species that produce the protein and so the role of each species.	Low amount of proteins in the soil sample/difficulties in the protein extraction and purification due to contaminant molecules present in soil. Problems with species attribution/necessity of an in-house database produced by NGS analysis.
Metabolomics	Targeted or untargeted metabolomics can be used to measure changes in specific metabolite levels in response to a given treatment. Detailed information regarding the produced metabolite.	Difficulties to attribute the species that produce the identified metabolite. Difficulties in the purification of the metabolites present in soil and the quantification.

**Table 2:** Some examples of field trials using transgenic Rhizobia.

Bacterium	Method of Application	Test Country	References
*Ensifer meliloti*	Alfalfa spray inoculation	USA	[188]
*E. meliloti*	Alfalfa seed coating	Ireland, Spain	[189,190]
*Bradyrhizobium japonicum*	Soybean seed coating	USA	[188]
*Rhizobium etli*	Bean seed coating	USA	[191]
*Rhizobium leguninosarum*	Liquid seed coating	France	[192]
*Rhizobium galegae*	Liquid seed coating	Finland	[193]

## Data Availability

Not applicable.
